# The Serum Resistome of a Globally Disseminated Multidrug Resistant Uropathogenic *Escherichia coli* Clone

**DOI:** 10.1371/journal.pgen.1003834

**Published:** 2013-10-03

**Authors:** Minh-Duy Phan, Kate M. Peters, Sohinee Sarkar, Samuel W. Lukowski, Luke P. Allsopp, Danilo Gomes Moriel, Maud E. S. Achard, Makrina Totsika, Vikki M. Marshall, Mathew Upton, Scott A. Beatson, Mark A. Schembri

**Affiliations:** 1 Australian Infectious Diseases Research Centre, The University of Queensland, Brisbane, Queensland, Australia; 2 School of Chemistry and Molecular Biosciences, The University of Queensland, Brisbane, Queensland, Australia; 3 Queensland Brain Institute, The University of Queensland, Brisbane, Queensland, Australia; 4 Faculty of Medicine and Dentistry, University of Plymouth, Plymouth, United Kingdom; MicroTrek Incorporated, United States of America

## Abstract

*Escherichia coli* ST131 is a globally disseminated, multidrug resistant clone responsible for a high proportion of urinary tract and bloodstream infections. The rapid emergence and successful spread of *E. coli* ST131 is strongly associated with antibiotic resistance; however, this phenotype alone is unlikely to explain its dominance amongst multidrug resistant uropathogens circulating worldwide in hospitals and the community. Thus, a greater understanding of the molecular mechanisms that underpin the fitness of *E. coli* ST131 is required. In this study, we employed hyper-saturated transposon mutagenesis in combination with multiplexed transposon directed insertion-site sequencing to define the essential genes required for *in vitro* growth and the serum resistome (i.e. genes required for resistance to human serum) of *E. coli* EC958, a representative of the predominant *E. coli* ST131 clonal lineage. We identified 315 essential genes in *E. coli* EC958, 231 (73%) of which were also essential in *E. coli* K-12. The serum resistome comprised 56 genes, the majority of which encode membrane proteins or factors involved in lipopolysaccharide (LPS) biosynthesis. Targeted mutagenesis confirmed a role in serum resistance for 46 (82%) of these genes. The murein lipoprotein Lpp, along with two lipid A-core biosynthesis enzymes WaaP and WaaG, were most strongly associated with serum resistance. While LPS was the main resistance mechanism defined for *E. coli* EC958 in serum, the enterobacterial common antigen and colanic acid also impacted on this phenotype. Our analysis also identified a novel function for two genes, *hyxA* and *hyxR*, as minor regulators of O-antigen chain length. This study offers novel insight into the genetic make-up of *E. coli* ST131, and provides a framework for future research on *E. coli* and other Gram-negative pathogens to define their essential gene repertoire and to dissect the molecular mechanisms that enable them to survive in the bloodstream and cause disease.

## Author Summary

The emergence and rapid dissemination of new bacterial pathogens presents multiple challenges to healthcare systems, including the need for rapid detection, precise diagnostics, effective transmission control and effective treatment. *E. coli* ST131 is an example of a recently emerged multidrug resistant pathogen that is capable of causing urinary tract and bloodstream infections with limited available treatment options. In order to increase our molecular understanding of *E. coli* ST131, we developed a high-throughput transposon mutagenesis system in combination with next generation sequencing to test every gene for its essential role in growth and for its contribution to serum resistance. We identified 315 essential genes, 270 of which were conserved among all currently available complete *E. coli* genomes. Fifty-six genes that define the serum resistome of *E. coli* ST131 were identified, including genes encoding membrane proteins, proteins involved in LPS biosynthesis, regulators and several novel proteins with previously unknown function. This study therefore provides an inventory of essential and serum resistance genes that could form a framework for the future development of targeted therapeutics to prevent disease caused by multidrug-resistant *E. coli* ST131 strains.

## Introduction

*Escherichia coli* O25b:H4-ST131 (*E. coli* ST131) is a recently emerged, globally disseminated clone that is often multidrug resistant and is responsible for a high proportion of community- and nosocomially-acquired urinary tract and bloodstream infections [1]–[6]. *E. coli* ST131 strains are also capable of causing complicated infections including acute pyelonephritis, osteomyelitis, septic arthritis and septic shock [7], [8]. *E. coli* ST131 are commonly associated with production of the CTX-M-15 enzyme, currently the most widespread extended spectrum β-lactamase (ESBL) of its type in the world [1], [9]. In addition to resistance against oxyimino-cephalosporins (i.e. cefotaxime, ceftazidime), and monobactams, *E. coli* ST131 strains are often co-resistant to fluoroquinolones [3], [10]. Indeed, most fluoroquinolone-resistant *E. coli* strains belong to a recently emerged and dominant subgroup of ST131 strains [11]. Some *E. coli* ST131 strains have also been reported to produce carbapenemases [12]–[14], thus severely limiting treatment options that are currently available against this clinically predominant clone [15].

*E. coli* ST131 strains, like many other uropathogenic *E. coli* (UPEC) strains, are derived from phylogenetic group B2 [3]. Typically, UPEC strains possess a large and diverse range of virulence factors that contribute to their ability to cause urinary tract and bloodstream infections, including adhesins, toxins, siderophores and protectins [15], [16]. Several studies have demonstrated that *E. coli* ST131 strains possess a similar suite of virulence factors and cause invasive disease, leading to the hypothesis that the widespread pathogenic success of *E. coli* ST131 strains may be in part due to enhanced virulence [7], [8], [17]. However, it has become clear from recent studies that *E. coli* ST131 strains do not possess a heightened virulence potential compared to other UPEC or B2 *E. coli* strains in causing invasive infections [18] or infections in nematodes and zebrafish embryos [19]. Thus, other factors such as enhanced metabolic capacity have been proposed to contribute to the fitness and pathogenic success of this dominant clone [20], [21].

The genome sequence of one of the best-characterized *E. coli* ST131 strains, EC958, was recently described [22]. *E. coli* EC958 is a member of the pulsed-field gel electrophoresis (PFGE) defined UK epidemic strain A, which represents one of the major pathogenic lineages (PFGE strains A–E) of ESBL producing *E. coli* causing urinary tract infections (UTI) across the UK [23]. *E. coli* EC958 is resistant to eight antibiotic classes, including oxyimino-cephalosporins, fluoroquinolones and sulphonamides. *E. coli* EC958 colonizes the bladder of mice in a type 1 fimbriae-dependent manner [22], can invade into bladder epithelial cells and form intracellular bacterial communities, can establish both acute and chronic UTI [24] and can inhibit the contraction of ureters, *in vitro* [25].

The ability to resist the bactericidal activity of serum, and thus survive in the bloodstream, represents an essential virulence trait for UPEC and other extra-intestinal *E. coli* (ExPEC) strains, including *E. coli* ST131 [26]–[28]. In *E. coli*, several mechanisms have been shown to contribute to serum resistance. The importance of O-antigens and K capsules in resistance to serum has been recognized since the 1960s and 1980s, respectively [29]–[31]; and their multiple types, combinations and length contribute differently to serum resistance [32]–[35]. The major outer membrane protein OmpA [36], plasmid-encoded proteins TraT [37], [38] and Iss [39], and the phage membrane protein Bor [40] have also been reported to contribute to serum resistance in *E. coli*. Notably, each of these resistance mechanisms has been studied in isolation and in different strain backgrounds. Thus, while serum resistance is clearly a complex phenotype determined by multiple elements, little is known about the combination of factors that contribute to resistance in a single strain.

High-throughput transposon mutagenesis combined with genome-wide targeted sequencing was used recently to study the essential genes in *Salmonella enterica* serovar Typhi and *Caulobacter crescentus* [41], [42]. Langridge *et al.* also used their transposon directed insertion-site sequencing (TraDIS) method to assay every gene for its role in the survival of *S.* Typhi in the presence of bile salts [42]. Similar approaches (INSeq, HITS, Tn-seq) have also been applied to a range of organisms to study gene requirements for survival in particular niches [43]–[46]. Here, we adapted TraDIS and designed a multiplexing method to define the essential genes required for *in vitro* growth (i.e. Luria-Bertani agar media supplemented with 30 µg/ml Cm at 37°C) and the serum resistome (i.e. genes required for resistance to human serum, in *E. coli* EC958). We show that the essential gene list of *E. coli* EC958 comprises 315 genes, 231 of which are shared with *E. coli* K-12. We also define for the first time a comprehensive inventory of genes required for resistance to human serum. Our study provides a molecular blueprint for understanding the mechanisms employed by *E. coli* ST131 to survive, grow in the bloodstream and cause disease.

## Results

### Application of multiplexed TraDIS to the *E. coli* ST131 strain EC958

**Figure 1:**
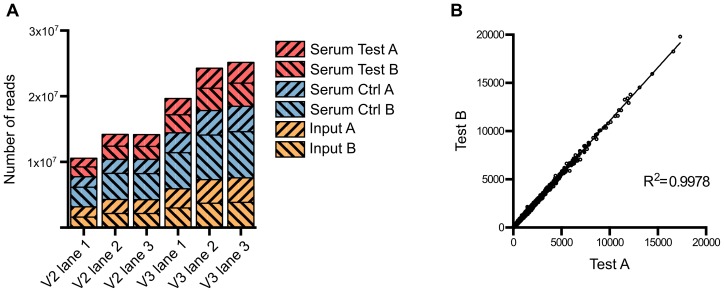
Summary of sequence read data from multiplexed TraDIS. (A) Number of tagged reads obtained in each lane of TruSeq version 2 and version 3 flowcells. (B) Correlation between the two biological replicates. The number of insertions in each gene from Test A was plotted against that from Test B; the correlation coefficient R^2^ indicates very low variation between the two replicates.

Approximately 1 million mutants were generated in the *E. coli* ST131 strain EC958 [22] using an in-house miniTn*5* transposon carrying a chloramphenicol (Cm) resistance gene derived from the pKD3 plasmid [47]. A primer comprising four functional regions was designed to facilitate specific sequencing of the transposon insertion sites on the Illumina HiSeq 2000 platform while allowing for intra-lane multiple sample indexing (Figure S1). This primer contained (5′-3′): (i) the P5 sequence to bind to TruSeq flowcells, (ii) the Illumina read 1 sequencing primer binding site, (iii) a 6-bp index sequence for multiple sample barcoding and (iv) a 25-bp transposon specific sequence designed to amplify the last 12 bp of the transposon and its adjacent genomic sequence. Using this custom primer, we successfully sequenced the transposon insertion sites for 6 samples on both TruSeq version 2 and version 3 flowcells (Figure 1A). Each sample yielded from 6.8 million to 15 million reads that were tagged with transposon specific sequence, 71% of which were reliably mapped to EC958 draft chromosome (excluding unscaffolded contigs and plasmids) (Table 1). All experiments were performed in duplicate, with the correlation coefficient for the number of insertions per gene for each pair of samples close to 1 (R^2^>0.99) and thus demonstrating a high level of reproducibility for each experiment (Figure 1B).

**Table 1:** Summary of sequencing and mapping results of multiplexed TraDIS runs.

	Tagged reads	Reads mapped to EC958 chromosome (%)	Unique insertion sites in EC958
Input A & B	16,281,107	11,558,154 (70.99)	502,068
Test A	6,819,691	4,879,098 (71.54)	438,706
Test B	7,572,730	5,408,136 (71.41)	436,858
Ctrl A	8,350,687	5,918,766 (70.87)	394,521
Ctrl B	15,006,139	10,727,954 (71.49)	485,459

### Essential genes in *E. coli* EC958

We initially used our saturated random insertion mutant library to determine the ‘essential genes of EC958’, defined as the set of genes required for growth on LB agar supplemented with Cm 30 µg/ml. We extracted genomic DNA (in duplicates: input A and B) directly from the library pool and sequenced using our multiplexed TraDIS protocol. We combined the reads from input A and B to maximize the coverage resulting in 16 million transposon-tagged reads, of which 11 million uniquely mapped to the EC958 chromosome, resulting in 502,068 unique insertion sites. This equates to an average of one insertion site every 9.92 bp, with a very low probability of having 100 consecutive bp without interruption by chance (*P* = 4.2×10^−5^).

The essential gene list was identified using a statistical analysis similar to that described by Langridge *et al.*, which recognized two distinct distributions of insertion indexes (number of insertions divided by gene length) for non-essential genes (gamma) and essential genes (exponential) and called those with insertion indexes less than or equal to the intercept of the two distributions as essential [42]. In our data, an insertion index cut-off of 0.0158, resulted in the identification of 315 genes as essential (Table S1). This cut-off is equivalent to a log_2_-likelihood-ratio (LLR) of −3.6, which means that our essential genes are at least 12 times more likely to belong to the exponential distribution (essential) than the gamma (non-essential) distribution.

**Figure 2:**
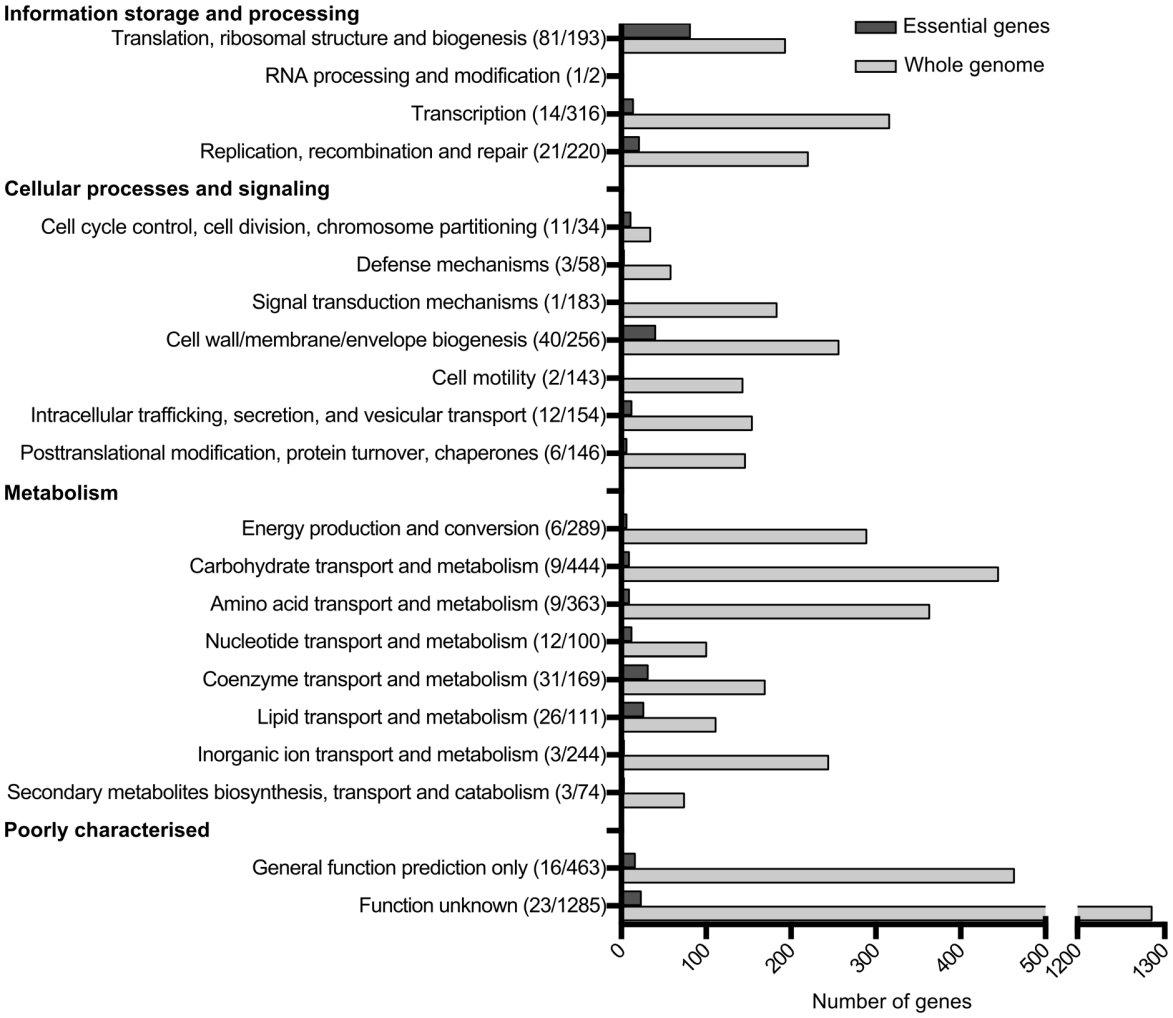
Number of essential genes in each COG functional category.

The functional category of each gene was identified based on the COG (Clusters of Orthologous Groups) numbers from the EC958 annotation (accession number PRJEA61443). Figure 2 shows an overview of essential functions in EC958 compared with the total number of genes in each functional category. Genes involved in translation, ribosomal structure and biogenesis account for 25% of the total number of essential genes in EC958, which is 42% of the total number of genes in this category. The second most abundant category in the essential gene list comprised genes involved in cell wall/membrane/envelope biogenesis (12%), followed by genes involved in coenzyme transport and metabolism. There were 23 essential genes with functions not identified in the COG database.

To investigate the conservation of EC958 essential proteins among different *E. coli* pathotypes, we performed tfastx alignment (FASTA v36) between the essential protein sequences and translated DNA from 50 *E. coli* complete genomes (Table S2). There were 270 (86%) proteins conserved across all genomes investigated. An additional 17 proteins were also present in more than 90% of the genomes. Only 6 proteins were specific for EC958 (not found in 50 genomes) (Table S3).

### The serum resistome of *E. coli* EC958

**Figure 3:**
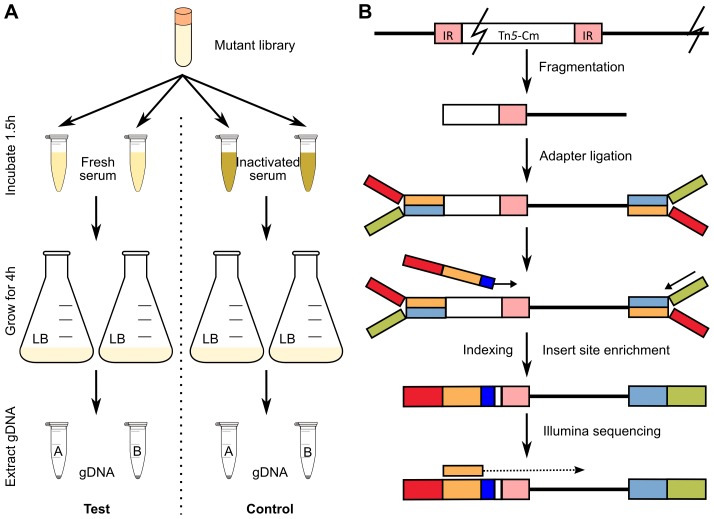
Experimental design to identify serum resistance genes in EC958. (A) Selection steps employed using fresh serum as test and inactivated serum as control. (B) Schematic illustration of the Illumina sequencing procedure including the use of a custom oligo for indexing and enrichment of insert sites.

Saturated transposon mutagenesis in combination with next-generation sequencing is a powerful tool for whole genome, high-throughput identification of all candidate genes involved in a particular phenotype. Here, we used our transposon mutant library in combination with TraDIS to identify genes from EC958 involved in resistance to human serum, thus enabling us to define the serum resistome of EC958. We designed a mutant selection procedure in which 1 million mutants were exposed to pooled fresh human serum for 90 minutes and then allowed to grow in LB broth for 4 hours before genomic DNA extraction. This procedure permitted the growth of serum resistant mutants while eliminating or inhibiting mutants that were sensitive to serum (Figure 3A). The procedure was performed in parallel with control samples where fresh serum was replaced by inactivated serum that lacked bactericidal activity (data not shown). The genomic DNA from test and control samples were sequenced using our modified Illumina multiplexed TraDIS procedure (Figure 3B) to generate multiple datasets (Figure 1A) that were analysed by the Bioconductor package edgeR after filtering out genes identified as essential [48].

**Figure 4:**
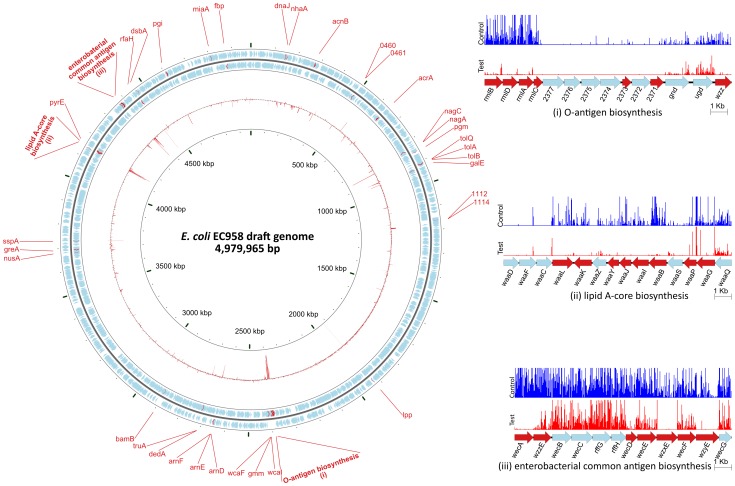
Overview of the *E. coli* EC958 serum resistance genes. The circular diagram depicts the location of 56 serum resistance genes on the *E. coli* EC958 genome. The two outer rings containing blue and red arrows illustrate the CDS and serum resistance genes, respectively, on the forward and reverse strand of the genome. The inner red ring represents the logFC between the control and the test samples for each CDS. The three insets represent a close-up look at three regions on the genome with the graphs showing the location and relative number of each mutant found in the control (blue) and test (red) samples.

The serum resistance genes were identified as genes that have significant reduction in read counts in the test samples compared to the control samples (i.e. less mutants survived after serum treatment) (Table S4). A stringent threshold of log_2_ fold change of read counts (logFC) less than −1 and an adjusted p-value less than 0.001 was used to identify significant genes that are involved in serum resistance (Figure S2). Figure 4 shows the names and genomic locations of the 56 genes that satisfied these stringent criteria.

Twenty-two (39.3%) of the genes belong to three operons responsible for LPS biosynthesis (including both O-antigen biosynthesis and lipid A-core biosynthesis) and enterobacterial common antigen (ECA) biosynthesis. This result represents the first layer of evidence demonstrating the importance of the O25 antigen as well as ECA in an *E. coli* ST131 background for protection from the bactericidal activity of human serum. Detailed characterisation of the O25 antigen gene cluster is discussed in subsequent sections. ECA is common to all *Enterobacteriaceae* and is expressed in both serum resistant (smooth) and sensitive (rough) strains, except for rough strains that are defective in the shared biosynthesis pathway affecting both O-antigen and ECA [49], [50]. Seven out of 12 genes in the ECA operon were required for serum resistance as determined by the TraDIS technique.

The remaining 60.7% of genes in the serum resistome identified by TraDIS included genes encoding lipoprotein, membrane proteins, regulators and hypothetical proteins (Table 2). Some of these also affect LPS such as *rfaH* (EC958_4322), encoding a known regulator required for LPS biosynthesis [51], [52] and virulence of pathogenic *E. coli* strains [53], [54], whilst others represent genes that have not previously been shown to be associated with serum resistance. The murein lipoprotein gene *lpp* (EC958_1897) showed the greatest difference between the test and control samples (logFC of −10), followed by *pgm* (EC958_0806), encoding phosphoglucomutase. Four hypothetical proteins were also identified, two of which (EC958_0460 and EC958_0461) were further characterized in this study (see below).

**Table 2:** Genes required for serum resistance identified by multiplexed TraDIS.

EC958 locus_tag	Gene Name	Matched in MG1655	Product	Serum^a^	LPS^c^	SDS MIC^d^ (%)	NaCl MIC^d^ (M)	COG code
EC958_0147	*dnaJ*	b0015	DnaJ chaperone	1	WT	WT	WT	O
EC958_0152	*nhaA*	b0019	Sodium/proton antiporter	0^b^	WT	WT	WT	P
EC958_0260	*acnB*	b0118	aconitate hydratase 2/2-methylisocitrate dehydratase	1	WT	WT	WT	C
EC958_0460	*0460*	-	-	3	X	WT	WT	-
EC958_0461	*0461*	-	-	1^b^	X	WT	WT	TK
EC958_0602	*acrA*	b0463	multidrug efflux system	N/A	N/A	N/A	N/A	M
EC958_0784	*nagC*	b0676	Transcriptional regulator, repressor of N-acetylglucosamine	1	WT	WT	WT	KG
EC958_0785	*nagA*	b0677	N-acetylglucosamine-6-phosphate deacetylase	3	X	0.063	WT	G
EC958_0806	*pgm*	b0688	Phosphoglucomutase	4	X	0.016	WT	G
EC958_0856	*tolQ*	b0737	membrane spanning protein in Tol-Pal cell envelope complex	3	WT	0.031	WT	U
EC958_0858	*tolA*	b0739	membrane anchored protein in Tol-Pal cell envelope complex	3	WT	0.031	WT	M
EC958_0859	*tolB*	b0740	periplasmic protein in Tol-Pal cell envelope complex	0	WT	WT	WT	U
EC958_0871	*galE*	b0759	UDP-galactose-4-epimerase	1	X	WT	WT	M
EC958_1112	*1112*	-	-	1	X	WT	WT	-
EC958_1114	*1114*	-	-	2	WT	WT	WT	-
EC958_1897	*lpp*	b1677	murein lipoprotein	6	WT	0.063	WT	M
EC958_2368	*wzz*	b2027	regulator of O-antigen chain length	1	X	WT	WT	M
EC958_2371	*2371*	-	-	2	LA	0.063	WT	R
EC958_2373	*2373*	-	-	N/A	N/A	N/A	N/A	E
EC958_2378	*rmlC*	b2038	dTDP-4-deoxyrhamnose-3,5-epimerase	4	LA	0.063	WT	M
EC958_2379	*rmlA*	b2039	glucose-1-phosphate thymidylyltransferase	2	X	WT	WT	M
EC958_2380	*rmlD*	b2040	dTDP-4-dehydrorhamnose reductase	4	LA	WT	WT	M
EC958_2381	*rmlB*	b2041	dTDP-glucose 4,6 dehydratase	3	X	WT	WT	M
EC958_2390	*wcaI*	b2050	glycosyl transferase involved in colanic acid synthesis	0^b^	WT	WT	WT	M
EC958_2391	*gmm*	b2051	GDP-mannose mannosyl hydrolase	1^b^	WT	WT	WT	F
EC958_2394	*wcaF*	b2054	predicted acyl transferase	4	X	0.063	0.5	R
EC958_2594	*arnD*	b2256	undecaprenyl phosphate-alpha-L-ara4FN deformylase	2	WT	0.063	WT	G
EC958_2596	*arnE*	b4544	ArnE/ArnF undecaprenyl-phosphate-α-L-Ara4N flippase	1	WT	WT	WT	P
EC958_2597	*arnF*	b2258	ArnE/ArnF undecaprenyl-phosphate-α-L-Ara4N flippase	2	WT	WT	WT	-
EC958_2652	*dedA*	b2317	conserved inner membrane protein	1^b^	WT	WT	WT	S
EC958_2653	*truA*	b2318	pseudouridylate synthase I	0^b^	WT	WT	WT	J
EC958_2822	*bamB*	b2512	lipoprotein required for OM biogenesis, in BamABCD complex	3	WT	0.063	WT	S
EC958_3571	*nusA*	b3169	transcription termination/antitermination L factor	0^b^	WT	WT	WT	K
EC958_3581	*greA*	b3181	transcript cleavage factor	2	WT	0.063	WT	K
EC958_3621	*sspA*	b3229	stringent starvation protein A	2	WT	0.063	WT	O
EC958_4029	*waaL*	b3622	O-antigen ligase	3	LA	0.063	WT	M
EC958_4030	*waaK*	b3623	Lipopolysaccharide core biosynthesis; heptosyl transferase IV	3	LA	0.063	WT	M
EC958_4032	*waaY*	b3625	Lipopolysaccharide core heptose (II) kinase	5	LA	WT	<0.3	T
EC958_4033	*waaJ*	b3626	UDP-glucose:(glucosyl)LPS α-1,2-glucosyltransferase	2	LA	0.063	WT	M
EC958_4034	*waaI*	b3627	UDP-D-glucose:(glucosyl)LPS α-1,3-glucosyltransferase	5	X	0.063	0.5	M
EC958_4035	*waaB*	b3628	UDP-D-galactose:(glucosyl)LPS-1, 6-D-galactosyltransferase	4	X	WT	WT	M
EC958_4037	*waaP*	b3630	Lipopolysaccharide core heptose (I) kinase	6	X	0.016	WT	-
EC958_4038	*waaG*	b3631	Lipopolysaccharide glucosyltransferase I	6	X	0.016	0.5	M
EC958_4050	*pyrE*	b3642	orotate phosphoribosyltransferase	2	WT	WT	WT	F
EC958_4246	*wecA*	b3784	undecaprenyl-phosphate α-N-acetylglucosaminyl transferase	5	LA	0.063	WT	M
EC958_4247	*wzzE*	b3785	ECA polysaccharide chain length modulation protein	2	X	0.063	WT	M
EC958_4252	*wecD*	b3790	TDP-fucosamine acetyltransferase	5	X	0.063	WT	-
EC958_4253	*wecE*	b3791	TDP-4-oxo-6-deoxy-D-glucose transaminase	2	WT	WT	WT	M
EC958_4254	*wzxE*	b3792	lipid III flippase	0^b^	WT	WT	WT	R
EC958_4255	*wecF*	b4481	TDP-Fuc4NAc:lipidIIFuc4NAc transferase	1^b^	WT	0.063	WT	-
EC958_4256	*wzyE*	b3793	ECA polysaccharide chain elongation	0^b^	WT	0.063	WT	-
EC958_4322	*rfaH*	b3842	DNA-binding transcriptional antiterminator	5	LA	0.016	<0.3	K
EC958_4336	*dsbA*	b3860	Protein disulfide oxidoreductase	1	WT	0.031	0.6	O
EC958_4480	*pgi*	b4025	glucosephosphate isomerase	1^b^	WT	0.063	WT	G
EC958_4658	*miaA*	b4171	Δ(2)-isopentenylpyrophosphate tRNA-adenosine transferase	0^b^	WT	0.063	WT	J
EC958_4725	*fbp*	b4232	fructose-1,6-bisphosphatase I	3	WT	WT	WT	G

a Serum sensitivity was shown as −log_10_ of viable count difference between t = 90 min and t = 0 min. The higher the number the more sensitive the mutant is.

b Serum sensitivity was determined by competitive assays: 0 means no difference was found between the wild-type and mutant; 1 means the mutant was more attenuated than the wild-type.

c LPS patterns were coded as followed: WT, similar to wild-type strain, no change observed; LA, only the band for lipid A-core observed; LA+1, lipid A-core band and only one band above it; X, changes observed, see Supplementary Figure S3 for band patterns.

d The wild-type (WT) MIC is 0.8 M for NaCl and 0.125% for SDS.

### Validation of serum resistance genes

As mentioned above, we employed a stringent threshold combining fold change and statistical significance to define the set of 56 genes in the EC958 serum resistome. In order to validate these findings, we attempted to test all 56 genes independently for their role in serum resistance. Using a modified lambda red mediated-homologous recombination approach [22], [47] we successfully generated defined knock-out mutants for 54 genes (96.4%) in EC958. We were unable to obtain mutants for the remaining 2 genes (*acrA* and EC958_2373) despite multiple attempts (Table 2).

The 54 defined mutants were subjected to serum susceptibility testing, whereby the number of surviving colonies after a 90-minute exposure to fresh pooled human serum was compared to the number of colonies prior to treatment (Table 2). A mutant was defined as susceptible to serum when its log difference was at least 1 (i.e. 10 fold reduction after exposure to serum). Forty-one genes (75.9%) contributed to serum resistance in EC958 using this assay. In the case of the remaining 13 mutants, it is possible that the lack of susceptibility to human serum was a reflection of the assay, suggesting that survival in serum within a mixed population of one million mutants may be very different from survival of a pure population carrying the same defective mutation. Therefore, to better mimic the condition of TraDIS library serum selection, a competitive assay was devised where EC958 wild-type was mixed equally with a mutant before exposure to serum and the competitive index of the mutant was measured. Using this competitive assay, five of the twelve mutants were significantly attenuated compared to the wild-type EC958 strain (Table 2). Thus, the overall number of validated susceptible mutants was 46 out of 54 tested (85.2%).

### Additional characteristics of serum resistant mutants

We hypothesized that one mechanism associated with enhanced sensitivity to human serum could be due to decreased membrane integrity caused by destabilization of the outer leaflet of the outer membrane. In order to test this, we examined the survival of the 54 mutants in response to outer membrane stresses (i.e. SDS) and osmotic potential (i.e. NaCl). In total, 50.0% (27/54) of the mutants displayed enhanced sensitivity to SDS and 11.1% (6/54) of the mutants displayed enhanced sensitivity to NaCl (Table 2). A comparative analysis of these phenotypes in the context of serum sensitivity is presented below.

### O25b antigen biosynthesis genes conferring resistance to serum

It is well established that O-antigen represents the main determinant for serum resistance in *E. coli*. However, there are more than 180 different O-antigens that have been defined in *E. coli* and these may contribute differently to serum resistance in individual bacterial strains [32], [33]. Furthermore, direct genetic evidence linking O-antigen biosynthesis to serum resistance is only available for a small number of specific O-antigen types. With this in mind, a detailed characterization of the O25b biosynthesis genes was performed using sequence comparison for function prediction in combination with analysis of LPS composition to deduce the role of each gene in resistance to serum.

**Figure 5:**
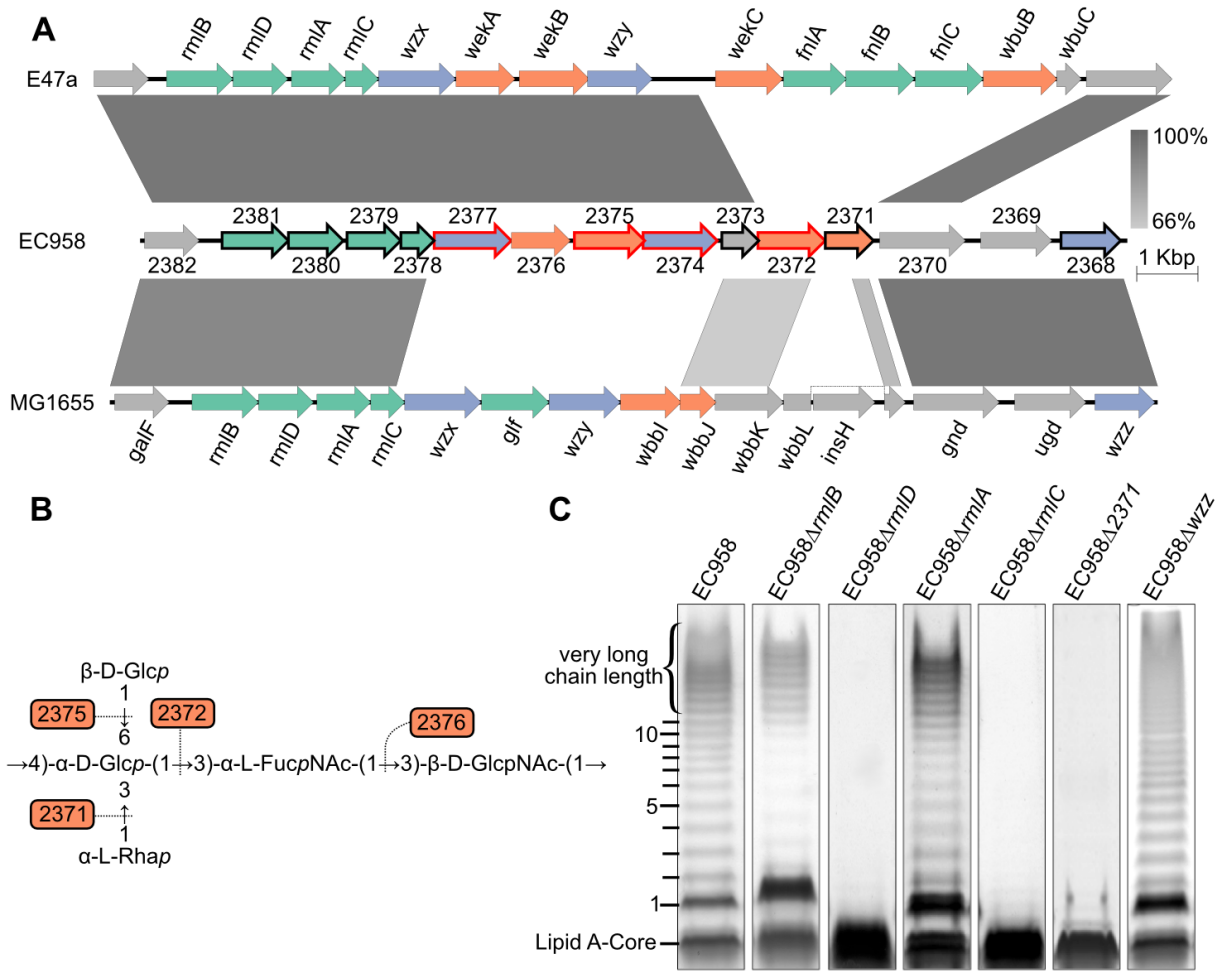
Characterization of O25b antigen genes in EC958. (A) Comparison of the O-antigen cluster in EC958 with MG1655 (NC_000913) and E47a (GU014554); genes in green are involved in sugar biosynthesis, genes in purple are involved in O-antigen processing and genes in orange encode glycosyl transferase enzymes; genes with a black outline were shown to be required for serum resistance and genes with a red outline were defined as essential. (B) Predicted function of the four glycosyl transferases in the biosynthesis of the O25b repeat unit [57], [58]. (C) LPS gels showing changes in O-antigen structures corresponding to mutations in each gene.

Similar to most *E. coli* strains, the O-antigen biosynthesis operon is located between the *galF* and *gnd* genes in EC958. Figure 5A shows a comparison of the EC958 O-antigen operon with the equivalent operon from the K-12 strain MG1655 and the O25 serotype *E. coli* strain E47a [32], [55].

#### Nucleotide sugar biosynthesis genes

The four genes encoding dTDP-α-L-rhamnose biosynthesis enzymes were highly conserved in the three strains, consistent with the presence of α-L-rhamnose in the O16 and O25 antigen repeat unit expressed by MG1655 and EC958, respectively [56]–[58]. The dTDP-α-L-rhamnose biosynthesis pathway starts from α-D-glucose-1-phosphate to produce dTDP-α-L-rhamnose via the catalysis of RmlA (to make dTDP-α-D-glucose), RmlB (to make dTDP-4-dehydro-6-deoxy-α-D-glucose), RmlC (to make dTDP-4-dehydro-6-deoxy-β-mannose) and RmlD (to make dTDP-α-L-rhamnose), respectively. LPS gel analysis of EC958 mutants lacking one of these four enzymes showed that RmlC and RmlD were required for the biosynthesis of O-antigen and mutation in either gene resulted in cells with only the lipid A core (Figure 5C). On the contrary, the LPS patterns of *rmlA* and *rmlB* mutants showed only a small change in intensity but retained most of the O-antigen chain length distribution, except for the very long chain length band. This might be explained by the existence of RffH and RffG, two isozymes of RmlA and RmlB, in EC958. RffH and RffG, however, were not able to fully compensate for RmlA or RmlB in resistance to serum (Table 2). The inability to produce the very long chain length O-antigen in *rmlA* and *rmlB* mutants might be a crucial factor in determining their susceptibility to human serum. Only the *rmlC* mutant displayed altered sensitivity to SDS, while mutations in *rmlABCD* did not affect sensitivity to NaCl (Table 2).

The O-antigen operon in E47a has another set of nucleotide sugar biosynthesis genes (*fnlABC*) for the biosynthesis of UDP-N-acetyl-α-L-fucosamine (UDP-FucNAc) from UDP-*N*-acetyl-α-D-glucosamine (UDP-GlcNAc). Curiously, EC958 does not have these three genes in the operon or anywhere else in its genome.

#### O-antigen processing genes

The O-antigen flippase (Wzx, EC958_2377) and O-antigen polymerase (Wzy, EC958_2374) from EC958 are highly similar to the corresponding genes from E47a. In contrast, both genes share very low similarity to the corresponding genes from MG1655. Mutation of the *wzx* gene in MG1655 results in the accumulation of high levels of the UndPP-O unit in the cytoplasm [59] and hypersensitivity to several antibiotics and other agents including nalidixic acid, tetracycline, mitomycin C and hydrogen peroxide [60]. Transcriptional analyses of MG1655 responses to the broad-spectrum biocide polyhexamethylene biguanide suggested that Wzx (also known as RfbX) might be involved in cellular stress responses [61]. In contrast to the previous studies, the *wzx* gene was defined as essential in this study (Figure 4, Table S1). This might be explained by the fact that Cm was used to select for transposon mutants and the *wzx* mutants might be hypersensitive to this antibiotic. The *wzy* O-antigen polymerase gene (EC958_2374) was also defined as essential in EC958 and thus was not further characterized. Mutation of the chain length regulator gene (*wzz*, EC958_2368) showed a non-modal distribution of O-antigen chain length in its LPS pattern (Figure 5C), an observation consistent with previous study describing the role of this gene [62]. The reduction of long chain length O-antigen in this mutant is likely to account for the serum sensitivity shown in our assay (Table 2).

#### Glycosyltransferases genes

Glycosyltransferases (GTs) are required to form the glycosidic bonds between sugars in an O-antigen repeat unit. There are now more than 100,000 GTs within 94 families (http://www.cazy.org). Based on the structure of the O25 repeat unit [63], [64], we predicted a requirement for 4 GTs in EC958. Bioinformatic analysis (blastp) confirmed that there are indeed 4 GTs within the O-antigen operon: EC958_2371, EC958_2372, EC958_2375 and EC958_2376. Surprisingly, two of the predicted GTs in EC958 are very different from those in E47a. Whether this is related to the different source of α-L-FucNAc in EC958 due to the lack of *fnlABC* remains to be investigated. Since the O25 antigen structure is known, we attempted to predict the glycosidic link formed by each GT using a combination of sequence similarity and O-antigen structure comparison as previously described [64]; the results of which are shown in Table 3 and Figure 5B. We were only able to mutate EC958_2371, while EC958_2372 and EC958_2375 were defined as essential in this study. Analysis of our EC958_2371 mutant confirmed that EC958_2371 is required for O-antigen biosynthesis (Figure 5C), serum resistance and resistance to SDS (Table 2).

**Table 3:** Summary of *E. coli* EC958 O25b antigen biosynthesis genes.

Locus tag	Gene	No. of amino acids	Similar protein	% identities/% positives/no. of amino acid overlap	predicted function
EC958_2381	rmlB	361	dTDP-glucose 4,6-dehydratase [Escherichia coli NA114] (YP_006139244)	99/100/361	dTDP-glucose 4,6-dehydratase
EC958_2380	rmlD	299	dTDP-4-dehydrorhamnose reductase [Escherichia coli NA114] (YP_006139241)	100/100/299	dTDP-4-dehydrorhamnose reductase
EC958_2379	rmlA	292	glucose-1-phosphate thymidylyltransferase [Escherichia coli NA114] (YP_006139242)	100/100/292	glucose-1-phosphate thymidylyltransferase
EC958_2378	rmlC	180	dTDP-4-dehydrorhamnose 3,5-epimerase [Escherichia coli NA114] (YP_006139241)	99/99/180	dTDP-4-dehydrorhamnose 3,5-epimerase
EC958_2377	wzx	419	O-antigen flippase Wzx [Escherichia coli E47a] (ADI43260)	95/98/149	O-antigen flippase
EC958_2376	wekA	321	putative glycosyl transferase family 2 [Escherichia coli ABU83972] (ADN46883)	93/98/321	glycosyl transferase family 2; α-L-Fuc*p*NAc-(1->3)-β-D-Glc*p*NAc
EC958_2375	wekB	382	putative glycosyl transferase [Escherichia coli E47a] (ADI43262)	93/97/382	glycosyl transferase family 4; β-D-Glcp-(1->6)- α-D-Glcp
			glycosyl transferase WcmS [Escherichia coli E1020-72, O158] (ADN43874)	39/59/372	
EC958_2374	wzy	405	O-antigen polymerase Wzy [Escherichia coli E47a] (ADI43263)	92/95/346	O-antigen polymerase
EC958_2373	wbbJ	201	predicted lipopolysaccharide biosynthesis O-acetyl transferase [Escherichia coli str. K-12 substr. MG1655] (NP_416537)	68/80/194	predicted lipopolysaccharide biosynthesis O-acetyl transferase
EC958_2372	wbuH	366	Putative glycosyltransferase WbuH [Escherichia coli O4:K3:H5] (AAT85654)	29/49/372	glycosyl transferase family 4; α-D-Glcp-(1->3)- α-L-FucpNAc
EC958_2371	wbbL	263	Rhamnosyltransferase [Escherichia coli str. K-12 substr. MG1655] (P36667)	60/76/260	glycosyl transferase family 2; Rhamnosyltransferase:α-L-Rha*p*(1->3)α-D-Glc*p*
EC958_2370	gnd	468	6-phosphogluconate dehydrogenase [Escherichia coli NA114] (YP_006137974.1)	100/100/468	6-phosphogluconate dehydrogenase
EC958_2369	ugd	388	UDP-glucose-6-dehydrogenase [Escherichia coli NA114] (YP_006137975)	100/100/388	UDP-glucose-6-dehydrogenase
EC958_2368	wzz	325	regulator of length of O-antigen component of lipopolysaccharide chain [Escherichia coli NA114] (YP_006137976)	100/100/325	O-antigen chain length regulator

### Other serum resistance mechanisms affecting LPS in EC958

LPS gel analysis was performed on all 54 defined mutants to identify genes that contribute to serum resistance by affecting LPS (Figure S3). The normal LPS pattern of EC958 consists of 12 bands including a thick bottom band representing the lipid A-core and an 11-band laddering pattern of lipid A-core bound O-antigen polymers, followed by approximately 6 thick bands of very long O-antigen chain length (Figure 5C). In addition to the 6 genes involved in O-antigen biosynthesis mentioned above, the LPS patterns of 20 mutants were altered in comparison to wild-type EC958; 6 of these mutants (*waaLKYJ*, *wecA* and *rfaH*) only produced a lipid A-core (Table 2 and Figure S3).

#### LPS core biosynthesis genes

The genes involved in biosynthesis of the LPS outer core in EC958 share strong similarity with those from K-12 MG1655 (*waaL* and *waaGPBIJYK*) [reviewed in 65], [66], suggesting that the LPS outer core structure in EC958 is the same as that in MG1655. As expected from the functions of *waaGBIJK* in MG1655, the *waaG* mutant showed the smallest size of lipid A-core, indicating that its whole outer core was not linked to the lipid A-inner core. Mutations in *waaIJYK* also produced an expected LPS pattern of lipid A-core only, as these mutations prevent the linkage of O-antigen to the outer core. The LPS of the *waaB* and *waaP* mutants produced O-antigen laddering patterns containing an abnormal lipidA-core band, indicating that although the core structures were changed, these changes still allow the linking of O-antigen to the outer core. WaaL is responsible for the ligation of O-antigen to the outer core and mutation of this gene resulted in the synthesis of a lipid A-core without O-antigen. The EC958 *waaL* and *waaGPBIJYK* mutants were sensitive to human serum and SDS (except for the *waaB* and *waaY* mutant), while only the *waaG*, *waaI* and *waaY* mutants displayed enhanced sensitivity to NaCl (Table 2).

#### ECA biosynthesis genes

Seven serum resistant candidate genes within the ECA biosynthesis *wec* operon were identified by TraDIS (Table 2). Two of these genes (*wzxE* and *wzyE*) were not confirmed by serum assays to confer resistance and no changes were observed in the LPS patterns of these two mutants (Table 2, Figure S3). The *wzyE* mutant (but not the *wzxE* mutant) was, however, more sensitive to SDS (Table 2). The LPS patterns of the *wzzE* mutant revealed changes in the lipid A-core and in the modulation of O-antigen chain length (Figure S3). WzxE has previously been shown to preferentially form a protein complex with WzyE and WzzE for the biosynthesis of ECA over Wzy and Wzz [67]. However, our results suggested that WzzE might also contribute to the chain length regulation of O-antigen in EC958. WecA has been shown previously to be involved in the biosynthesis of O7, O18, O75, and O111 antigen [68] and our results indicate that the same is also true for O25b, thus explaining the role of WecA in serum resistance in EC958. The *wecD* mutant was highly sensitive to serum, possessed an LPS profile that was altered in both the lipid A-core and the amount of O-antigen, and displayed enhanced sensitivity to SDS (Table 2, Figure S3). Further investigation is required to understand the involvement of WecD in lipid A-core and O-antigen biosynthesis. Finally, none of the ECA biosynthesis mutants were altered in sensitivity to NaCl.

#### Other genes

The remaining 8 mutants with altered LPS patterns were all confirmed as serum sensitive (Table 2). They included one mutant, *rfaH*, which produced only lipid A-core, and 7 mutants with different O-antigen patterns: *hyxA* (EC958_0460), *hyxR* (EC958_0461), *nagA* (N-acetylglucosamine-6-phosphate deacetylase), *pgm* (phosphoglucomutase), *galE* (UDP-galactose-4-epimerase), EC958_1112 and *wcaF* (predicted acetyltransferase involved in colanic acid synthesis [69]). Some of these genes were also associated with resistance to additional stresses; EC958 *rfaH* and *wcaF* mutants were sensitive to SDS and NaCl, while *nagA* and *pgm* mutants were sensitive to SDS (Table 2).

### Novel O-antigen chain length regulators

**Figure 6:**
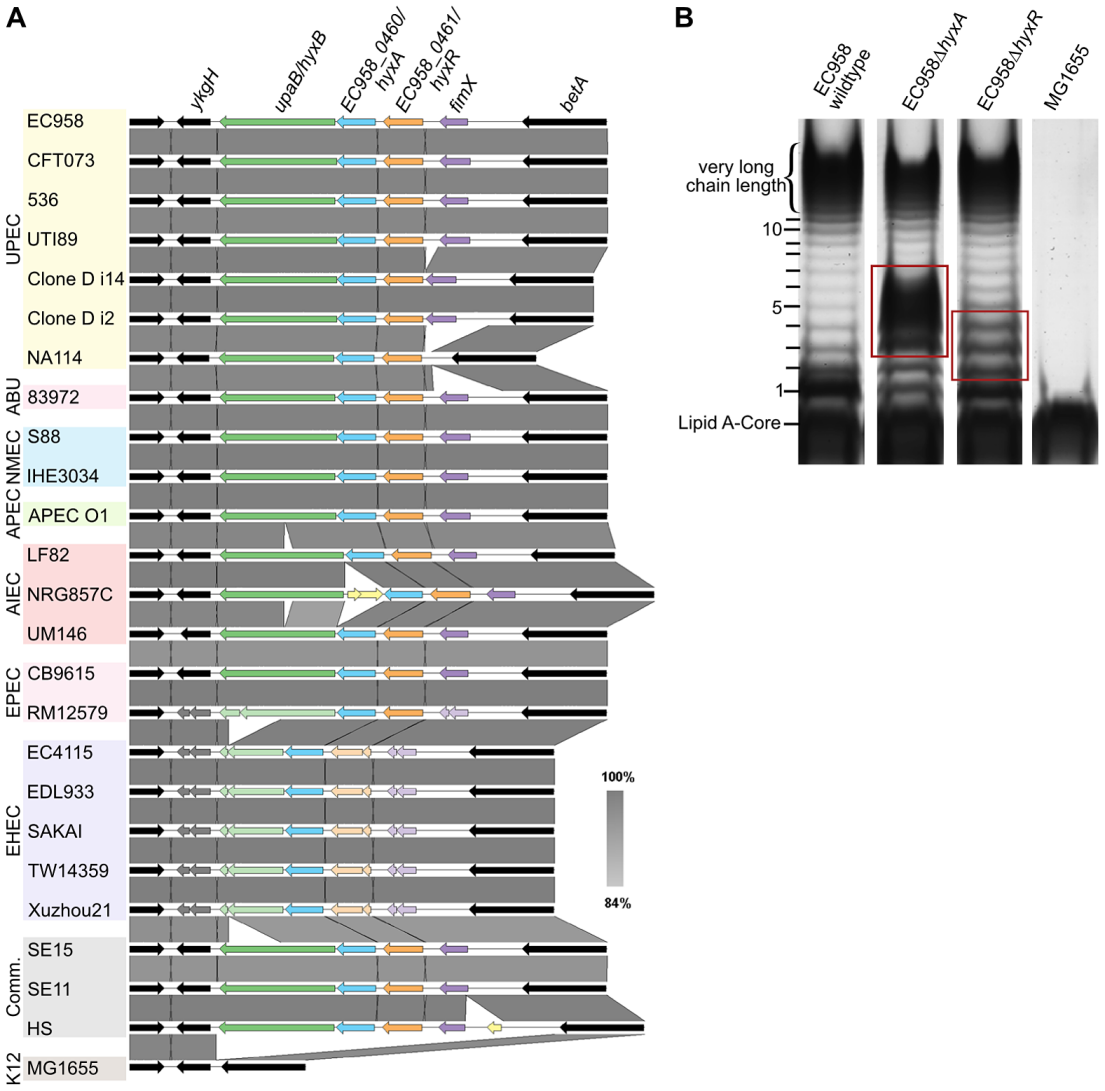
Genetic context of the *hyxA* and *hyxR* genes and their role in O-antigen synthesis. (A) The conserved location of PAI-X containing *hyxAR* genes in *E. coli* genomes of various pathotypes. (B) The LPS pattern of the *hyxA* and *hyxR* mutants and their complemented strains, demonstrating a role for the *hyxA* and *hyxR* genes in O-antigen chain length regulation. Red boxes highlight differences in LPS patterns compared with that of the wild-type.

Mutation of the *hyxA* and *hyxR* genes in EC958 resulted in the modulation of O-antigen chain length (Figure 6B). The EC958 *hyxA* mutant exhibited an increased proportion of O-antigen chain of 2 to 6 units with maximum number at 3–5 units and reduction in very high chain length polymer. The EC958 *hyxR* mutant had an increased proportion of O-antigen polymer of 2–4 units. The *hyxA* and *hyxR* genes are located in a pathogenicity island (PAI-X) consisting of 4 genes (*fimX* and *hyxRAB*) as previously described in the UPEC strain UTI89 [70]. The *hyxB* gene (EC958_0459) has also been named *upaB* due to its function as an autotransporter [71], and we prefer to maintain this nomenclature. This island is present (in several variations) in 24 out of 50 *E. coli* genomes across all pathotypes (Figure 6A) and current sequence data suggest that it is exclusive to *E. coli*.

No functional prediction was found for *hyxA*, and thus this is the first report of *hyxA* involvement in serum resistance by regulating O-antigen chain length. The *hyxR* gene encodes a LuxR-like response regulator that suppresses the nitrosative stress response and contributes to intracellular survival in macrophages by regulating *hmpA*, which encodes a nitric oxide-detoxifying flavohaemoglobin [70]. The expression of the *hyxR* gene is regulated through bidirectional phase inversion of its promoter region by the upstream gene *fimX*, which encodes a tyrosine-like recombinase [70]. It is also worth noting that the contribution of *hyxA* to serum resistance was greater than *hyxR*, as demonstrated by the 3-log reduction in viability by the *hyxA* mutant compared to the *hyxR* mutant. A serum sensitive phenotype for the *hyxR* mutant was only observed in mixed competition assays, and both mutants did not exhibit altered sensitivity to SDS or NaCl (Table 2).

### Novel serum resistance mechanisms not affecting LPS

A major advantage of whole genome approaches such as TraDIS lies in their power of discovery. Out of 46 genes that define the serum resistome of EC958, 21 (46%) genes were confirmed to be required for serum resistance independent of altered LPS patterns (Table 2; Figure S3). The function of these genes ranged across 10 COG functional categories and included ‘Carbohydrate transport and metabolism’ (4 genes), ‘Cell wall/membrane/envelope biogenesis’ (3), ‘Posttranslational modification, protein turnover, chaperones’ (3) and many others (Table 2). Twelve of these genes were also associated with enhanced sensitivity to SDS and one with enhanced NaCl sensitivity (Table 2)

Of the non-LPS genes required for serum resistance, the most notable was *lpp* (logFC −10, log difference = 6) (Table 2). The *lpp* gene encodes one of the most abundant proteins in *E. coli* and is responsible for the stabilisation and integrity of the bacterial cell envelope [72]. Mutation of the *lpp* gene results in the formation of outer membrane blebs, leakage of periplasmic enzyme ribonuclease, decreased growth rate in media of low ionic strength or low osmolarity and hypersensitive to toxic compounds [73], [74]. Indeed, the EC958 *lpp* mutant was more sensitive to SDS, suggesting decreased membrane integrity (Table 2). To the best of our knowledge, this study is the first to show a direct link between Lpp and serum resistance.

Another set of genes notable in our TraDIS analysis include *tolQAB*, which encode three of the six proteins (YbgC-YbgF-TolQ-R-A-B-Pal) that make up the Tol-Pal system of *E. coli* cell envelope. The Tol-Pal system is responsible for maintaining the integrity of the outer membrane. TolQRA form an inner membrane complex in which TolQR is necessary for its stability [75]. TolB, a periplasmic protein, connects the inner membrane complex with the peptidoglycan-associated lipoprotein, Pal, which is anchored to the outer membrane [76]. In our study, mutation of the *tolA* and *tolQ* genes caused sensitivity to human serum and increased sensitivity to SDS, while the *tolB* mutant did not (Table 2). Our results demonstrate that the Tol-Pal system is important for resistance to human serum, and thus describe a novel function for this important cell wall complex.

BamB is a lipoprotein that is part of the BamABCD complex. Mutation in *bamB* results in increased outer membrane permeability, thus enhancing sensitivity to rifampin and dramatically reducing growth on SDS and novobiocin [77]. Our data showed that mutation of the *bamB* gene in EC958 resulted in increased sensitivity to both human serum and SDS (Table 2).

Our TraDIS experiment also indicated that the modification of lipid A with L-Ara4N was important for serum resistance. Three (*arnDEF*) of the seven genes involved in the biosynthesis and attachment of L-Ara4N to lipid A-core were identified as part of the serum resistome of EC958 and their role was confirmed by mutagenesis (Table 2). This mechanism is known to confer resistance to polymixin B by preventing its binding to lipid A [Reviewed in 78], [79], [80]. ArnD catalyzes a deformylation step to generate UDP-L-Ara4N before it is transported across the inner membrane by ArnEF [79], [81]. The requirement of ArnDEF for serum resistance indicates that EC958 requires L-Ara4N modification to evade the antimicrobial activity of cationic peptides present in human serum. Interestingly, only the *arnD* mutation conferred sensitivity to SDS (Table 2), which might suggest a role of UDP-L-Ara4N in maintaining membrane integrity. Further investigation is needed to understand why ArnT, the final enzyme required for transferring the L-Ara4N residue to the 4′-phosphate group of lipid A-core, was not identified in our TraDIS-defined serum resistome.

We also identified three genes encoding catabolic enzymes that contributed to the serum resistance phenotype of EC958 (*gmm*, *pgi* and *fbp*) and confirmed their role by mutagenesis (Table 2). Of these genes, only the *pgi* mutant displayed enhanced sensitivity to SDS (Table 2). Gmm is a GDP-mannose mannosyl hydrolase capable of hydrolyzing both GDP-mannose and GDP-glucose [82]. This enzyme contributes to the biosynthesis of GDP-fucose, a component of colanic acid, possibly by influencing the concentration of GDP-mannose or GDP-glucose in the cell and thus regulating cell wall biosynthesis [82]. Both Pgi and Fbp catalyze the production of D-fructose-6-phosphate from β-D-glucose-6-phosphate and fructose-1,6-bisphosphate, respectively [83], [84]. D-fructose-6-phosphate is a precursor for the biosynthesis of UDP-GlcNAc, which in turn is required for peptidoglycan, lipid A and ECA biosynthesis. Thus, these three enzymes may catalyse key reactions that, if disrupted, could adversely affect the downstream biosynthesis of cell surface components including colanic acid, peptidoglycan, lipid A and ECA.

### An *ompA* mutant is protected from serum killing when present at a low proportion in a mixed bacterial population

**Figure 7:**
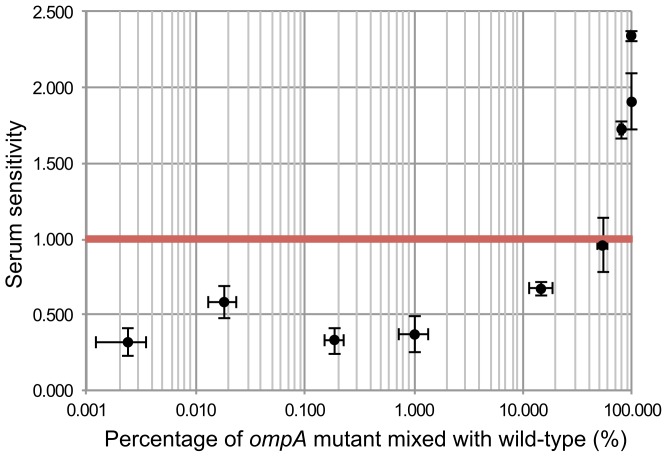
*In trans* complementation of wild-type EC958 protects *ompA* mutants from serum killing. Wild-type EC958 and an EC958 *ompA* mutant were mixed at different ratios, exposed to human serum for 90 minutes, and the EC958 *ompA* mutant was examined for its sensitivity to human serum (expressed as the −log_10_ CFU difference between time 90 and time 0). The red line indicates the threshold below which a strain was considered resistant to serum killing. The EC958 *ompA* mutant was protected from killing when present at a percentage of 15% or less in the mixed bacterial suspension.

Of all the chromosomal genes previously attributed to serum resistance, the only gene that was not identified in our TraDIS screen was *ompA*. To examine this further we constructed an EC958 *ompA* mutant and indeed observed it was sensitive to killing by human serum (Figure 7). One way to explain this discrepancy is that the phenotype of an *ompA* mutant could be complemented *in trans* by other *ompA*-intact bacteria in a mixed population such as the mutant library. In fact, OmpA inhibits serum-mediated killing by binding to C4b-binding protein (C4BP) to prevent the activation of C3b via the classical complement pathway [85], and OmpA is known to be released from *E. coli* cells when treated with serum [86]. In our mutant library, *ompA* mutants only accounted for approximately 0.02% of the total bacterial cells, and thus we hypothesized that the release of OmpA from 99.98% of the cells, when treated with serum, provided OmpA *in trans* to complement *ompA* mutants. We tested this hypothesis by mixing the *ompA* mutant with wild-type EC958 at various ratios and indeed showed that *ompA* mutants were protected from serum killing if the proportion of *ompA* mutants was less than 15% (Figure 7). This result strongly suggests that *in trans* complementation of OmpA prevents the identification of *ompA* as a serum resistance gene in our assay.

### Complementation of selected mutants restores serum resistance

To further demonstrate the function of non-LPS genes in serum resistance, we selected three orphan genes (*acnB*, *greA* and *fbp*) that do not belong to an operon to perform genetic complementation. For these experiments, the selected gene was amplified by PCR, cloned into the low copy number plasmid pSU2718G, and transformed into the respective mutant strain for complementation. In each case, the phenotype of the complemented strain exactly matched that of wild-type EC958 (Table 4). Taken together, these results confirm the role of *acnB*, *greA* and *fbp* in serum resistance and provide a further layer of evidence to support the use of techniques such as TraDIS in functional gene discovery.

**Table 4:** Complementation of selected mutants.

Strains	Phenotype		
	Serum^a^	SDS (%)^b^	NaCl (M)^b^
EC958 (wild-type)	0	0.125	0.8
EC958Δ*acnB*	1	0.125	0.8
EC958Δ*acnB* + pAcnB	0	0.125	0.8
EC958Δ*greA*	2	0.063	0.8
EC958Δ*greA* + pGreA	0	0.125	0.8
EC958Δ*fbp*	3	0.125	0.8
EC958Δ*fbp* + pFbp	0	0.125	0.8

a Serum sensitivity was shown as −log_10_ of viable count difference between t = 90 min and t = 0 min. The higher the number the more sensitive the mutant is.

b Minimal inhibitory concentration (MIC) of SDS (%) and NaCl (M).

## Discussion

The rapid advancement of new sequencing technologies has created novel opportunities to interrogate biological systems that were not previously possible. TraDIS was first described as a method that combined high-density mutagenesis with Illumina next generation sequencing technology to study the essential genes of *S.* Typhi and the conditional essential genes required for survival in bile [42]. The increased data output afforded by next generation DNA sequencing is particularly useful and cost-effective for small bacterial genomes, however it presents technical and bioinformatical challenges for applications such as TraDIS that utilize low complexity DNA libraries. Here we present the application of a modified version of TraDIS that is amenable to multiplexing using the Illumina HiSeq 2000 platform, and we demonstrate its effectiveness by using it to define the essential gene repertoire and the serum resistome of a multidrug resistant strain from the globally disseminated *E. coli* ST131 lineage.

The multiplexed TraDIS protocol utilizes a newly designed custom oligonucleotide in the library enrichment step of the Illumina library preparation protocol (Figure S1). This oligonucleotide incorporates the Illumina sequencing primer-binding site into transposon specific DNA fragments, enabling the use of the standard Illumina sequencing primer and eliminating the need to design and optimize another sequencing primer for each new transposon sequence. The 6-bp barcode immediately after the sequencing primer-binding site allows 12-sample multiplexing within one lane. The use of 12 barcodes at the first 6 nucleotides of read 1 increased the complexity of the library compared with the original TraDIS protocol, thus reducing data loss due to mis-identification of clusters [87], [88]. However, the number of useable reads from our sequencing runs was still low (15–20% of total reads). We believe non-specific amplification in the enrichment step was the main cause, and further optimization of the enrichment PCR conditions is required. Similar approaches combining transposon mutagenesis with high-throughput sequencing (Tn-seq [45], INSeq [43], HITS [44]) have also been used to address different scientific questions, including the identification of essential genes and genes associated with enhanced fitness in specific growth conditions [41], [42], [45], determination of niche-specific essential genes [43], [44], [89], identification of genes associated with tolerance to various agents/conditions [42], [90] and many other applications as reviewed elsewhere [91], [92]. In terms of insertion density, we achieved 502,068 independent insertion sites with a density of 1/10 bp (i.e. one insertion every 10 bp), which is comparable with the work by Christen *et al.* (1/8 bp in *C. crescentus*) [41], Langridge *et al.* (1/13 bp in *Salmonella* Typhi) [42] and Barquist *et al.* (1/9 bp in *Salmonella* Typhimurium) [46].

The identification of the essential gene set for a single organism is challenging due to several factors, including the presence of transposon insertion cold spots (i.e. regions of low transposon insertion frequency), the difficulty in distinguishing mutations that prevent growth from those that severely reduce growth rate, pre-existing gene duplications and the specific growth conditions used in the experiment [93]–[95]. In this study, we define essential genes as those genes that, when mutated by transposon insertion, either prevent or severely attenuate growth on LB agar media supplemented with 30 µg/ml Cm at 37°C. The cut-off value to determine whether a gene is essential was defined as the intercept of two distributions of the insertion index of each gene: the exponential distribution representing essential genes and the gamma distribution representing non-essential genes [42]. This means that our essential genes also include those genes that can tolerate insertions but were severely attenuated in the input pool. Out of 315 essential genes, 64 genes had no transposon insertions, 178 genes had 1 to 5 transposon insertions and 73 genes had more than 5 transposon insertions (Table S1).

The high density of insertion sites achieved in our study provided reliable data for the identification of essential genes within the EC958 genomes with a minimal probability of false positive calls due to transposon insertion cold spots. The identification of essential genes has previously been performed using several approaches in *E. coli* K-12 (strains MG1655 and W3110) [96], [97]. Baba *et al.* generated null mutations by lambda-red recombination in 3985 *E. coli* W3110 genes (the Keio library), but were unable to mutate 303 candidate essential genes [97]. This set of essential genes was further consolidated by manual literature review on the EcoGene website (www.ecogene.org), which reduced the set to 289 genes. Of the 315 essential genes identified for EC958 in this study, 231 genes (73%) matched those previously described in the EcoGene list (Table S1). There were 84 essential genes specific for EC958, twenty-four of which do not have homologs in the MG1655 genome. In contrast, 58 genes previously identified as essential for *E. coli* K-12 were either not present in EC958 or not identified in our analysis. The majority of essential genes in EC958 are conserved with 91% of the genes present in more than 90% of *E. coli* complete genomes available.

In this study, we provided two layers of evidence for the role of each serum resistance gene: by simultaneously assaying a large mutant library and by generation of defined mutants for independent phenotypic testing. Indeed, using defined mutagenesis we were able to confirm a role for 46 of the 56 genes identified by TraDIS in serum resistance. To the best of our knowledge, this represents the first large scale follow-up of TraDIS data in this manner and highlights the effectiveness of the technique in large-scale functional genomics. Our study also revealed that trans complementation of specific mutants can occur in a large mutant library population, as demonstrated by our findings with an *ompA* mutant. We also demonstrated complete complementation of mutants containing deletions in the *acnB*, *greA* and *fbp* genes, corroborating their novel role in serum resistance independent of LPS alterations. Finally, we provided further insight into the mechanistic action of the serum resistance genes identified in EC958 by examining the survival of the respective mutants to outer membrane stresses that affect antimicrobial access and osmotic potential.

The search for genetic determinants of serum resistance in bacteria has been ongoing since the 1970s [98]. Our current understanding of the mechanisms that promote bacterial resistance to human serum include a role for surface structures such as O antigens, K antigens, outer membrane proteins (OmpA), and plasmid-encoded proteins (TraT, Iss) [37]–[39]; notably, however, not all of these mechanisms are required for resistance in a single bacterial strain [32], [33]. Our study represents the first report to simultaneously investigate the entire serum resistome of one strain. Our results demonstrated that both the lipid A-core and O25 antigen are crucial for serum resistance in EC958, while K antigen does not contribute to serum resistance. Out of 54 defined mutants investigated, half had changes in their LPS gel patterns, all of which resulted in serum sensitivity. In contrast, none of the K capsular biosynthesis genes were identified in our TraDIS screen. This result is similar to that reported for the O75:K5 UPEC strain GR-12, where alterations in O75 LPS affected serum resistance more than a K5 null mutation [34]. It is likely, however, that there are strain-specific differences for the role of O antigen and K capsule in serum resistance, and that this reflects differences in the make-up of these structures. For example, previous analysis of an *E. coli* O4:K54:H5 blood isolate revealed that the K54 antigen contributes more to serum resistance than the O4 antigen [99]. Other K antigens such as the K1 and K2 capsules have also been shown to play an important role in serum resistance [100], [101]. The K antigen expressed by EC958 has not been typed but genomic analysis shows that EC958 has a group 2 capsular gene cluster that conforms to the conserved structure of this group [Reviewed in 102]. However, region 2, which encodes glycosyltransferases specific for each K type, shares such low similarity with available sequences in the GenBank database that deducing its K type *in silico* was not possible.

The O25 antigen gene cluster was further characterized using sequence analysis, targeted mutation and LPS profiling. All of the dTDP-α-L-rhamnose biosynthesis genes (*rmlCADB*) were required for serum resistance. However, EC958 lacks the biosynthesis genes for UDP-FucNAc, a component of O25 antigen unit. If, based on the cross reaction of antiserum against the O25 antigen with O25b expressing cells, we assume that EC958 has the same O-antigen repeat unit as the O25 determined from previous studies [57], [58], then EC958 must possess a novel mechanism for the synthesis or uptake of UDP-FucNAc.

Two additional surface antigens that contribute to serum resistance in EC958 are the enterobacterial common antigen and colanic acid (M antigen). Mutations in five ECA biosynthesis genes rendered EC958 susceptible to serum. While mutation of three of these genes (*wecA*, *wzzE* and *wecD*) affected LPS and sensitivity to SDS, mutation of *wecE* and *wecF* did not change LPS (although a *wecF* mutant was more sensitive to SDS), suggesting that the ECA may be involved in serum resistance, perhaps indirectly via its role in membrane integrity. Our data also suggest the involvement of colanic acid in serum resistance. Three genes encoding for colanic acid biosynthesis (*wcaI*, *gmm* and *wcaF*) were identified by TraDIS. Gmm is most likely to be involved in the biosynthesis of colanic acid [82], while mutation of the *wcaI* gene did not confer serum resistance. The product of *wcaF* was predicted to be an acetyltransferase [103] required for colanic acid production [69]. The EC958 *wcaF* mutant possessed an altered LPS pattern with a reduced amount of O-antigen (especially very long chain length O-antigen) and was sensitive to both SDS and high osmolarity. The enhanced sensitivity of the EC958 *wcaF* mutant could therefore be explained by a number of factors, including altered LPS, altered colonic acid and reduced overall membrane integrity.

A number of other genes were identified that contributed to serum resistance in an LPS-dependent manner. The gene *nagA* encodes N-acetylglucosamine-6-phosphate deacetylase, an enzyme important for the metabolism of N-acetyl-D-glucosamine [104]. It catalyzes the first step in producing UDP-GlcNAc, a nucleotide sugar required for ECA, lipid A and peptidoglycan biosynthesis, by deacetylating N-acetylglucosamine-6-phosphate to glucosamine-6-phosphate [Reviewed in 105]. However, NagA is not solely responsible for the production of UDP-GlcNAc because glucosamine-6-phosphate can also be obtained via GlmS from fructose-6-phosphate or taken up from the environment by ManXYZ [105]. Indeed, the LPS banding pattern of the *nagA* mutant was different to that of the parent strain (i.e. it possessed thicker second and third bands from the bottom of the gel; Figure S3), suggesting its enhanced sensitivity phenotype may be associated with a predominantly shorter O antigen.

Pgm is a phosphoglucomutase that catalyses the reversible conversion of glucose-1-phosphate to glucose-6-phosphate, an important step in galactose and maltose catabolism [106]. A *pgm* mutant has several phenotypes; it is defective in swimming and swarming mobility [107], it possesses an aberrant (shorter and wider) cell morphology, is sensitive to detergents [108] and it stains blue with iodine when grown in the presence of galactose [106]. An EC958 *pgm* mutant produced little full length O-antigen; the majority of its LPS condensed into a thick band of incomplete lipid A-core and a thin clear band of lipid A-core plus one unit of O-antigen. This feature is consistent with the high serum and SDS sensitivity phenotypes observed for this mutant.

GalE is a well-studied enzyme that catalyzes the interconversion of UDP-galactose and UDP-glucose [Reviewed in 109]. Both nucleotide sugars are required for colanic acid biosynthesis. Furthermore, UDP-glucose is used in three steps to synthesize the LPS outer core (catalyzes by WaaG, WaaI and WaaJ). LPS patterns of the *galE* mutant exhibited a very thick band of lipid A-core, suggesting that the lipid A-core in this strain has multiple sizes. This may be explained by the limiting effect of UDP-glucose in the three steps involved in its incorporation into the outer core. UDP-glucose can also be synthesized by GalU from glucose-1-phosphate [110], which may explain why an EC958 *galE* mutant could still make LPS (Figure S3). Despite being able to make LPS, however, the *galE* mutant was sensitive to human serum. Whether this sensitivity can be attributed to the effect a *galE* mutation has on LPS or colanic acid remains to be determined.

We have demonstrated the successful application of multiplexed TraDIS for a functional genomics study targeted at *E. coli* EC958, a prototype strain from the globally disseminated and multidrug resistant *E. coli* ST131 lineage. This approach enabled the first description of an essential gene set from an ExPEC strain. Our work has also defined the serum resistome in *E. coli* EC958. This comprehensive inventory of *E. coli* EC958 genes that contribute to this phenotype provides a framework for the future characterization of virulence genes in ExPEC as well as other Gram-negative pathogens that cause systemic infection.

## Materials and Methods

### Ethics statement

Approval for the collection of human blood was obtained from the University of Queensland Medical Research Ethics Committee (2008001123). All subjects provided written informed consent.

### Bacterial strains and growth conditions

*E. coli* EC958 was isolated from the urine of a patient presenting with community UTI in the Northwest region of England and is a representative member of the UK epidemic strain A (PFGE type), one of the major pathogenic lineages causing UTI across the United Kingdom [23]. EC958Δ*lac*, which contained a mutation in the *lac* operon, was used in competitive assays. This strain had an identical growth rate to wild-type EC958. Strains were routinely cultured at 37°C on solid or in liquid Luria Broth (LB) medium supplemented with the appropriate antibiotics (Cm 30 µg/ml or gentamicin 20 µg/ml) unless indicated otherwise.

### Generation of miniTn*5*-Cm mutant library

A miniTn*5*-Cm transposon containing a Cm cassette flanked by Tn*5* mosaic ends (sequence from Epicenter) was PCR amplified from pKD3 plasmid DNA (*Not*I digested) using primers 2279 5′- CTGTCTCTTATACACATCTcacgtcttgagcgattgtgtagg-3′ and 2280 5′- CTGTCTCTTATACACATCTgacatgggaattagccatggtcc-3′. The PCR reactions were performed using Phusion High-Fidelity DNA polymerase (New England BioLabs). The amplicon was purified using the QIAGEN MinElute PCR purification kit before being phosphorylated using T4 polynucleotide kinase (New England BioLabs) and subjected to the final purification step. A total of at least 800 ng of this miniTn*5*-Cm transposon DNA was incubated in an 8 µl reaction containing 4 µl of EZ-Tn*5* transposase (Epicenter Biotechnologies) at 37°C for 1 h then stored at −20°C.

Bacterial cells were prepared for electroporation as previously described [42]. Briefly, cells were grown in 2×TY broth to an OD_600_ of 0.3–0.5, then harvested and washed three times in 0.5× volume of 10% cold glycerol before being resuspended in a 1/1000× volume of 10% cold glycerol and kept on ice. A volume of 60 µl cells was mixed with 0.2 µl of transposomes and electroporated in a 2 mm cuvette using a BioRad GenePulser set to 2.5 kV, 25 µF and 200Ω. Cells were resuspended in 1 mL SOC medium and incubated at 37°C for 2 hours, then spread on LB agar plates supplemented with Cm 30 µg/mL. After incubation overnight at 37°C, the total number of colonies was estimated by counting a proportion from multiple plates. Chloramphenicol resistant colonies were resuspended in sterilised LB broth using a bacteriological spreader before adding sterile glycerol to 15% total volume and stored in −80°C. Each batch of mutants contained an estimated 32,000 to 180,000 mutants. The final library of 1 million mutants was created by pooling 11 mutant batches, resulting in a cell suspension of 2×10^11^ CFU/ml.

### Transposon library screening in human serum

Freshly pooled human serum was collected from at least two healthy individuals on the day of the experiment. Ten milliliters of blood was collected from each person and centrifuged at 4000 rpm for 10 minutes to collect the serum. Approximately 2×10^8^ viable mutants were incubated in 1 ml of 50% freshly pooled human serum in LB broth at 37°C for 90 minutes. The control samples were prepared the same way but were incubated with inactivated serum (Millipore) instead of fresh serum. Both control and test experiments were performed in duplicate. The cells were then washed twice with sterile 1×PBS to remove serum, transferred to 100 ml LB broth and allowed to grow at 37°C with 250 rpm shaking for 4 hours. The genomic DNA was then extracted from 5 ml of each culture using Qiagen 100-G genomic tips.

### Multiplexed TraDIS

Genomic DNA was standardized to 3.6 µg in a volume of 120 µl before being sheared by Covaris S2 according to the Illumina TruSeq Enrichment gel-free method (TruSeq DNA sample preparation v2 guide). The subsequent steps of DNA end repair, DNA end adenylation and adapter ligation were also done following the Illumina TruSeq v2 instructions. The adapter-ligated fragments containing transposon insertion sites were enriched using a transposon-specific indexing forward primer and the Illumina reverse primer Index 1 (Table S5) at 500 nM each per reaction. This 89 bp forward primer binds specifically to miniTn*5*-Cm transposon (25 bp) and carries 64 bp overhang which includes 6 bp index sequence, 33 bp binding site for Illumina read 1 sequencing primer and 23 bp P5 sequence for binding to the flowcell. The enrichment step was done using the KAPA Library Amplification kit (KAPA Biosystems) at an annealing temperature of 60°C for 22 cycles. The KAPA Library Quantification kit was used to measure the concentration of DNA fragments in the enriched library. Twelve libraries from 12 samples were pooled to equimolar concentrations to achieve a cluster density of 850 K/mm^2^ when 10 nM of library pool was loaded onto the flowcell. The 12-plex pool was loaded on 3 lanes of TruSeq v2 and 3 lanes of TruSeq v3 flowcells for sequencing using a 100 cycles, paired-end protocol to access the reproducibility and read quantity among lanes and flowcells. The data from six samples (Figure 1A) were presented in this study. The TraDIS sequence data from this study was deposited on the Sequence Read Archive (SRA) under the BioProject number PRJNA189704.

### Analysis of nucleotide sequence data

Sequence reads from the FASTQ files were split according to twelve 6 bp index sequences combined with the 37 bp transposon-specific sequence using fastx_barcode_splitter.pl (total length of 43 bp as barcodes, allowing for 2 mismatches) (FASTX-Toolkit version 0.0.13, http://hannonlab.cshl.edu/fastx_toolkit/index.html). The barcode matching reads were trimmed off the 43 bp barcode at the 5′end and 25 bp of potential low quality at the 3′ end, resulting in high quality sequence reads of 31 bp in length that were used to map to the EC958 chromosome (PRJEA61443) by Maq version 0.7.1 [111]. Subsequent analysis steps were carried out as previously described [42] to calculate the number of sequence reads (raw read counts) and the number of different insertion sites for every gene, which were then used to estimate the threshold to identify essential genes. The read counts and insertion sites were visualized using Artemis version 13.0 [112]. The circular genome diagram was generated by CGView [113] and linear genetic comparison was illustrated using Easyfig version 2.1 [114].

### Statistical analyses

We identified genes required for survival in human serum by comparing the differences in read abundance of each gene between the inactivated serum control and active serum test samples using the Bioconductor package edgeR (version 2.6.10) [48]. The raw read counts from two biological replicates of each treatment were loaded into the edgeR package (version 2.6.12) using the R environment (version 2.15.1). Genes that have very low read counts in all the samples (essential genes) were removed from further analysis. The composition bias in each sequence library was normalized using the trimmed mean of M value (TMM) method [115]. We then used the quantile-adjusted conditional maximum likelihood (qCML) for negative binomial models to estimate the dispersions (biological variation between replicates) and to carry out the exact tests for determining genes with significantly lower read counts in the test samples compared to the control samples [116], [117]. Stringent criteria of log fold-change (logFC) ≤−1 and false discovery rate ≤0.001 were chosen to define a list of the most significant genes for further investigation by phenotypic assays.

### Molecular methods

Chromosomal DNA purification, PCR and DNA sequencing of PCR products was performed as previously described [118]. Defined mutations were made using the λ-Red recombinase method with some modifications [22], [47]. In brief, the final PCR products were fused and amplified from three fragments containing two 500-bp homologous regions flanking the gene of interest and a Cm cassette from pKD3 plasmid (see Table S5 for list of primers). The fused PCR products were then electroporated into EC958 harbouring a gentamicin resistant plasmid carrying the λ-Red recombinase gene. Mutants were then selected and confirmed by sequencing. Complementation was done by cloning the gene of interest into a gentamicin resistant derivative of pSU2718 [119] at *Bam*HI-*Xba*I cut sites (primers listed in Table S5). The construct was then transformed into the respective mutant and induced using 1 mM IPTG before and during phenotypic assays.

### Serum resistance assay

Overnight bacterial cultures were washed in phosphate buffered saline (PBS) and then standardized to an OD_600_ of 0.8. Equal volumes (50 µL) of standardized cultures and pooled human sera were mixed and incubated for 90 min at 37°C (in triplicates). Viable counts were performed to estimate the number of bacterial cells prior to serum treatment (t = 0 min) and post serum treatment (t = 90 min). *E. coli* MG1655 was used as a control as it is completely killed by serum. Serum and PBS only samples served as sterility controls. Competitive serum resistance assays were performed in the same manner, except that a 50∶50 mixture of wild-type (EC958Δ*lac*) and mutant strains were used. Viable counts were performed on MacConkey agar, which allowed the differentiation of EC958Δ*lac* (non-lactose fermenter) and the mutant strains.

### SDS and NaCl sensitivity assays (MIC)

The MICs of SDS and NaCl were determined by broth microdilution method as previously described [120]. We used five concentrations for SDS including 0.125%, 0.0625%, 0.031%, 0.016% and 0.008% in LB. For NaCl, the range of concentration was 0.8 M, 0.6 M, 0.5 M, 0.4 M and 0.3 M.

### LPS gel assay

LPS was extracted from bacterial strains and LPS patterns were determined by Tricine-SDS Polyacrylamide gel electrophoresis (TSDS-PAGE) and visualized by silver staining as previously described [121], [122].

## Supporting Information

Figure S1 Diagram demonstrating the use of the custom primer with four functional regions for PCR enrichment of transposon derived fragments.
(PDF)

Figure S2 Results of edgeR analysis showing logFC of all genes and highlighting genes that satisfy the stringent cut-off for serum resistome.
(PDF)

Figure S3 LPS patterns of all defined mutants generated in this study. The LPS pattern of wild-type strain EC958 (WT) was used as reference to identify alteration in LPS of other mutants.
(PDF)

Table S1 Summary of transposon insertions in each CDS within the EC958 chromosome.
(XLSX)

Table S2 Sequence comparison of EC958 essential genes and genes from 50 *E. coli* complete genomes.
(XLSX)

Table S3 Essential genes unique to EC958.
(XLSX)

Table S4 Results of edgeR analysis comparing read counts between control and test samples to identify the serum resistome.
(XLSX)

Table S5 Primers used in this study.
(XLSX)
