# Supplementary figures and images for: The Serum Resistome of a Globally Disseminated Multidrug Resistant Uropathogenic Escherichia coli Clone
Source: PLoS Genet. 2013 Oct 3;9(10):e1003834. doi: 10.1371/journal.pgen.1003834 (PMC3789825; doi:10.1371/journal.pgen.1003834)

# Multiplexing using custom oligo

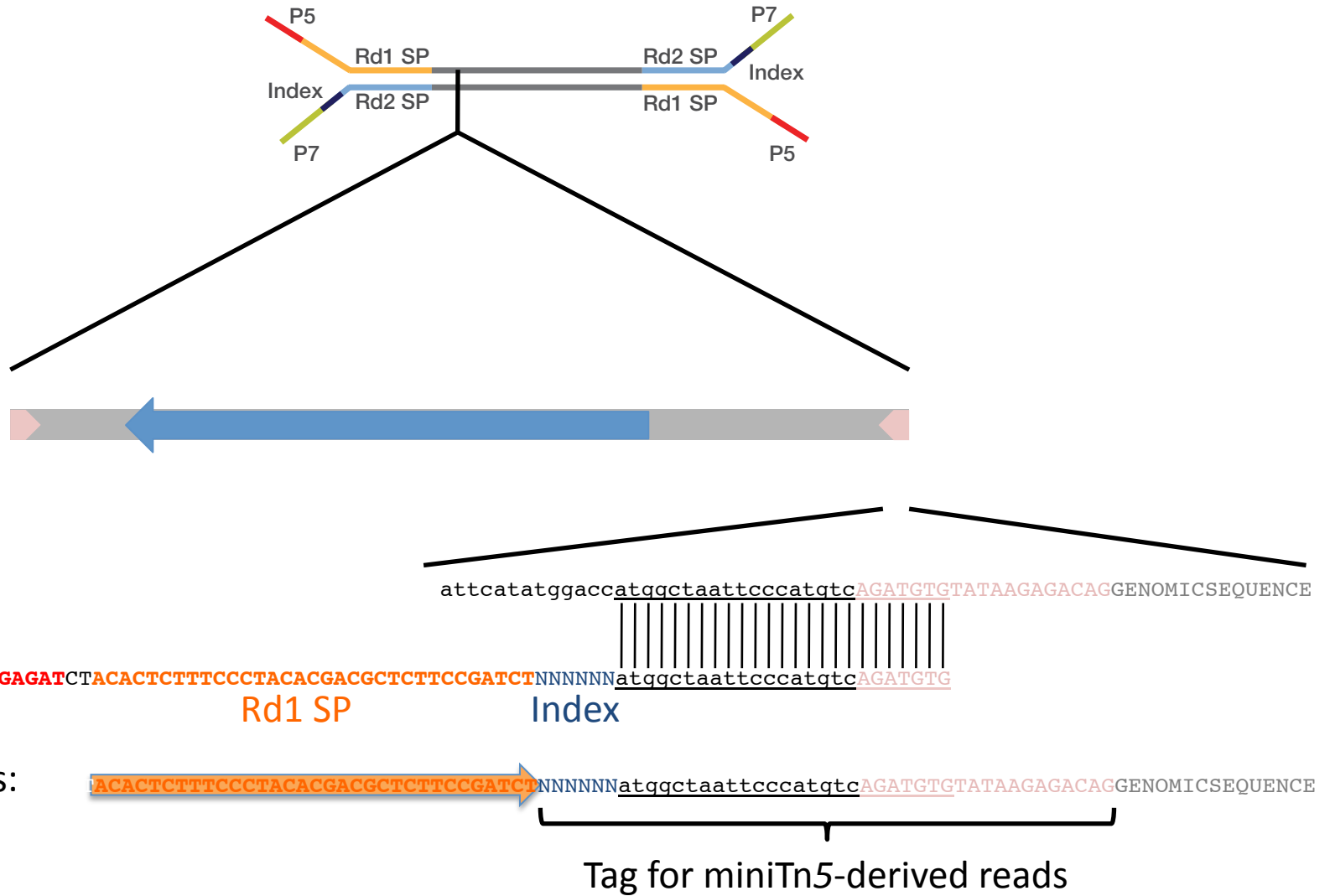

Supplement: Figure S1 — Diagram demonstrating the use of the custom primer with four functional regions for PCR enrichment of transposon derived fragments. (PDF) [file pgen.1003834.s001.pdf]

## Identification of serum resistance genes using edgeR

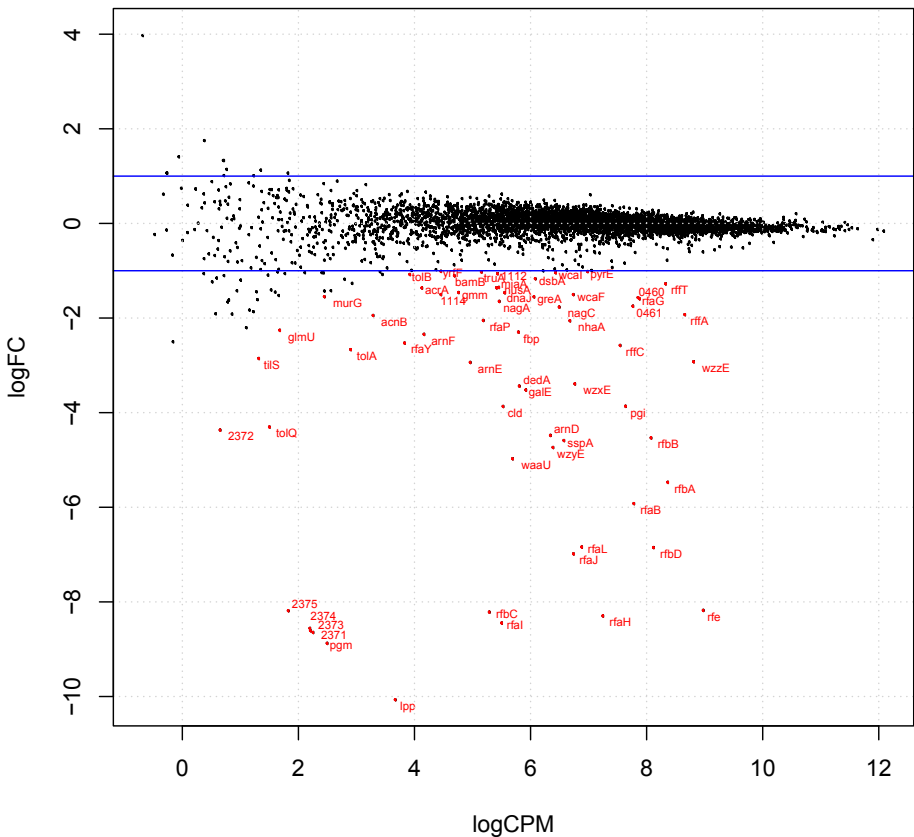

Supplement: Figure S2 — Results of edgeR analysis showing logFC of all genes and highlighting genes that satisfy the stringent cut-off for serum resistome. (PDF) [file pgen.1003834.s002.pdf]

Figure S3

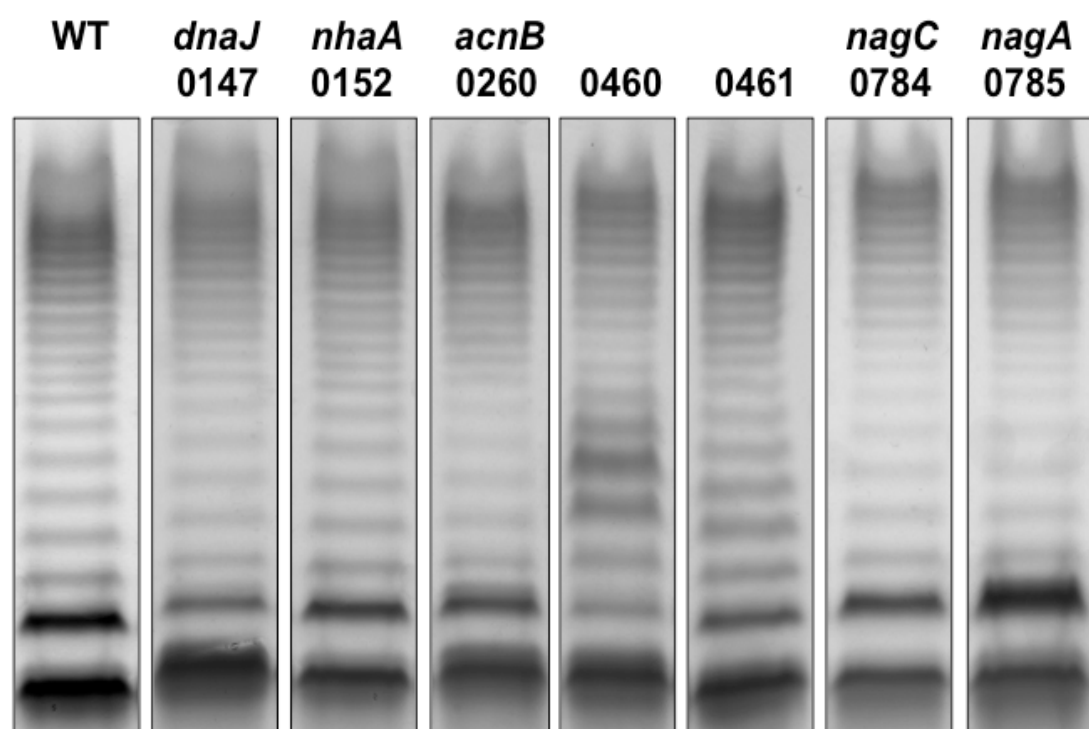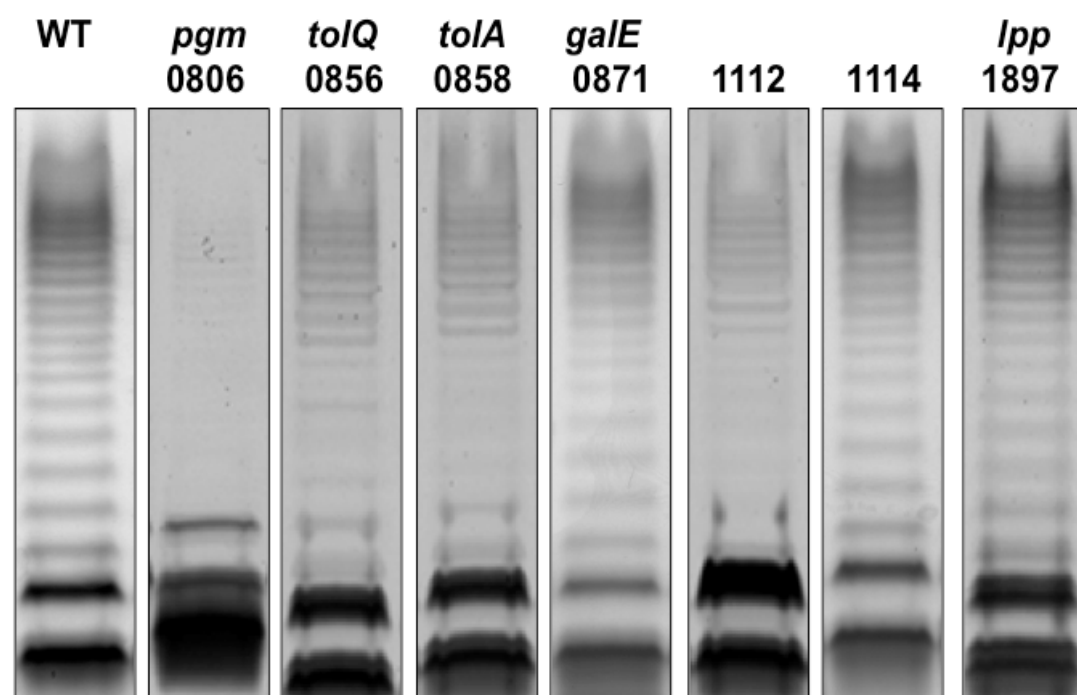

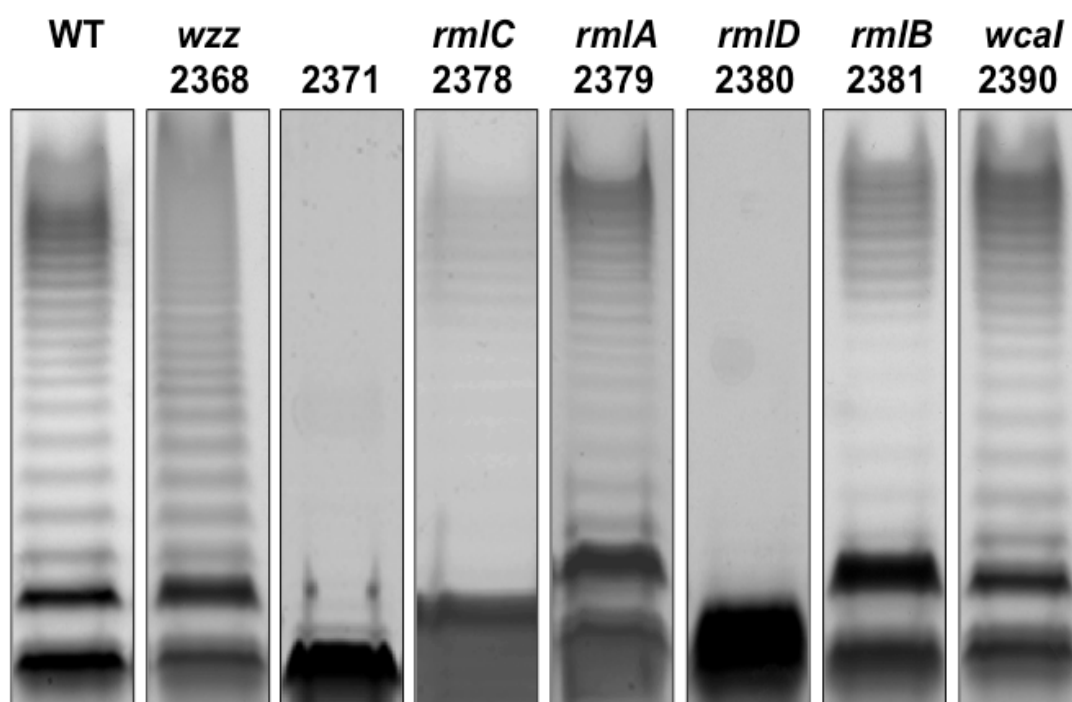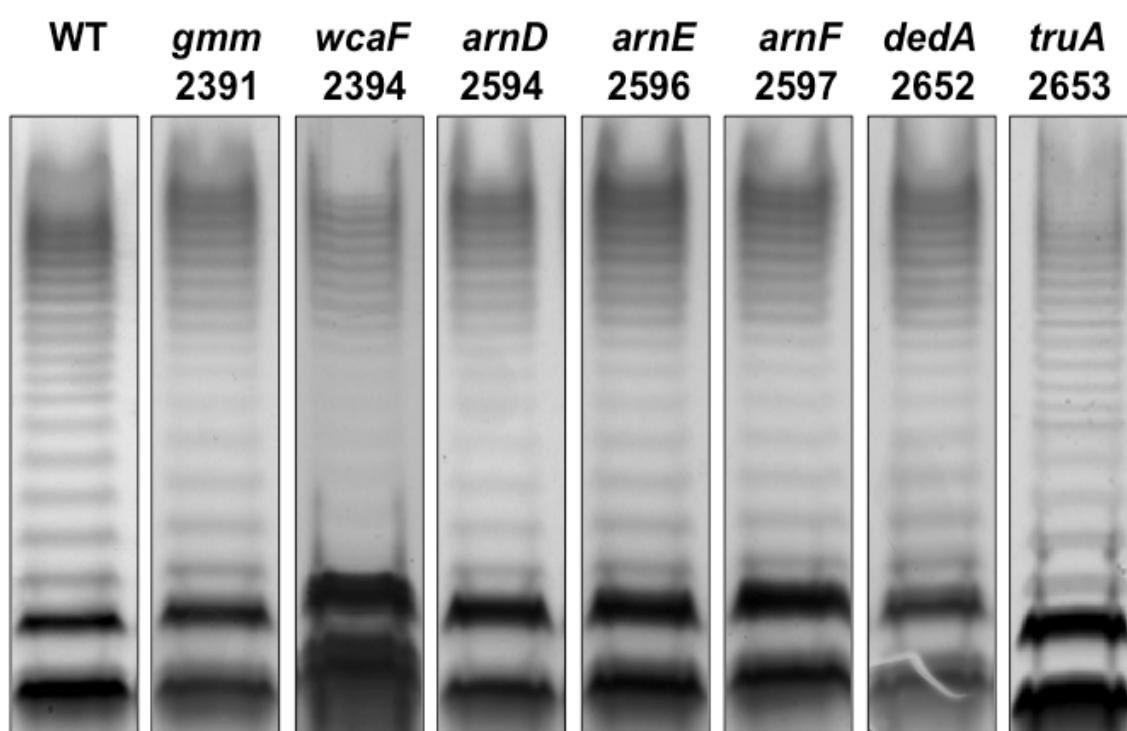

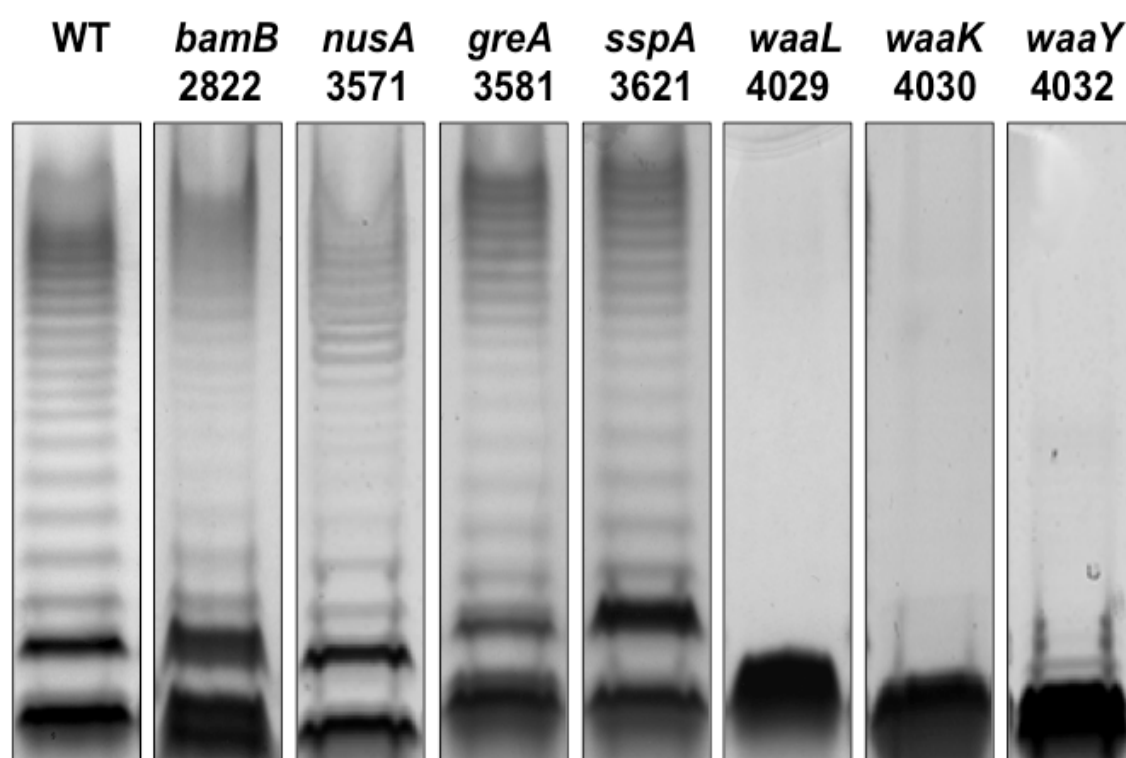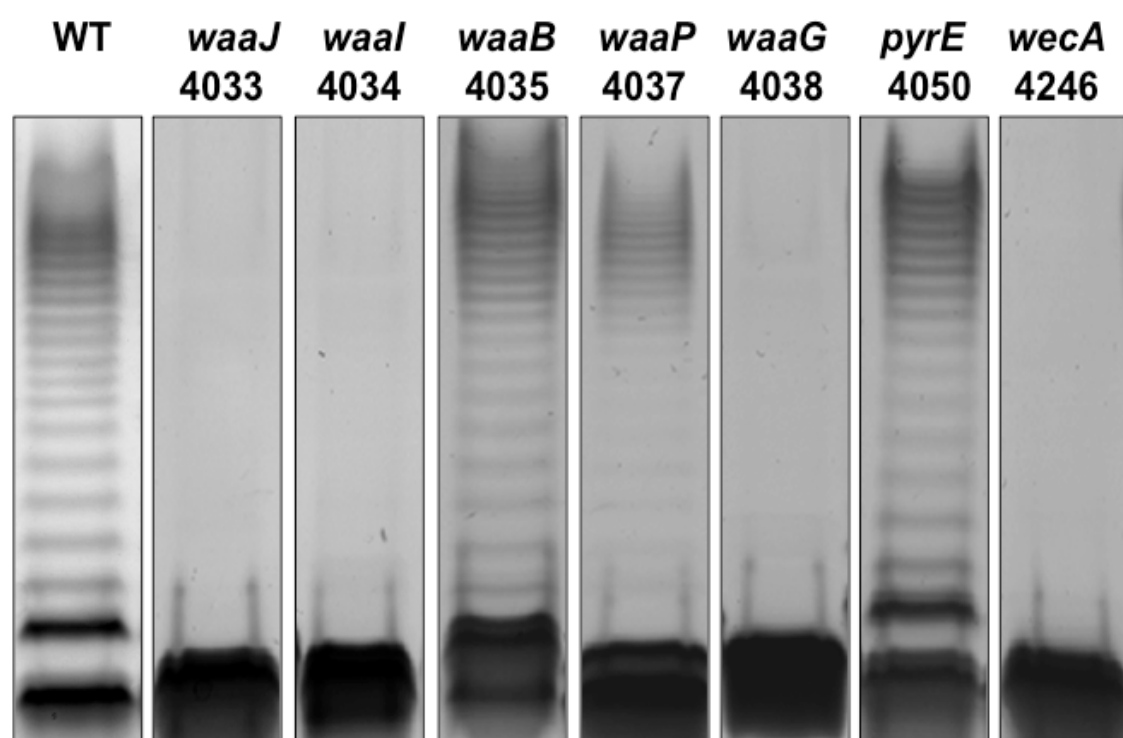

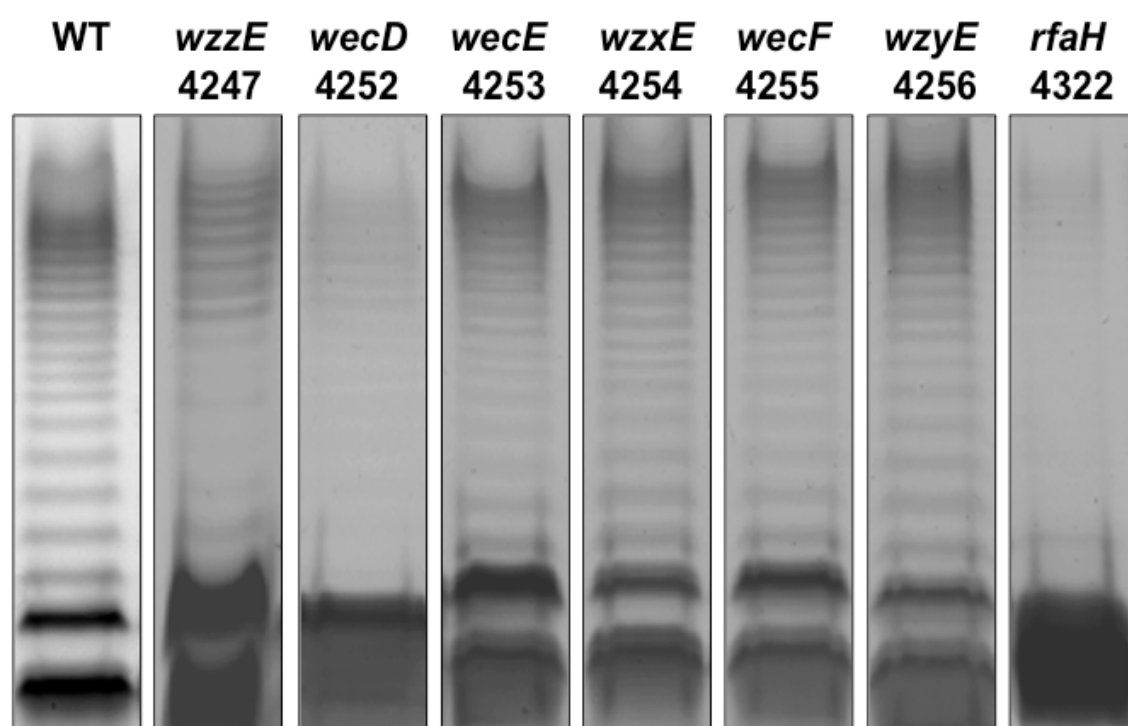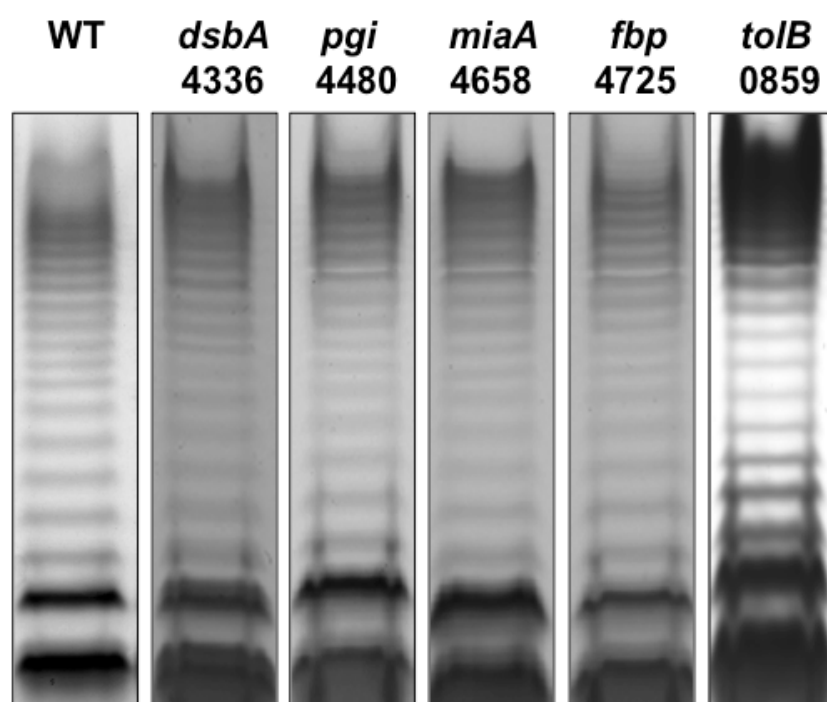

Supplement: Figure S3 — LPS patterns of all defined mutants generated in this study. The LPS pattern of wild-type strain EC958 (WT) was used as reference to identify alteration in LPS of other mutants. (PDF) [file pgen.1003834.s003.pdf]
